# A Chaperone-Assisted Degradation Pathway Targets Kinetochore Proteins to Ensure Genome Stability

**DOI:** 10.1371/journal.pgen.1004140

**Published:** 2014-01-30

**Authors:** Franziska Kriegenburg, Visnja Jakopec, Esben G. Poulsen, Sofie Vincents Nielsen, Assen Roguev, Nevan Krogan, Colin Gordon, Ursula Fleig, Rasmus Hartmann-Petersen

**Affiliations:** 1 Department of Biology, University of Copenhagen, Copenhagen, Denmark; 2 Lehrstuhl für Funktionelle Genomforschung der Mikroorganismen, Heinrich-Heine Universität, Düsseldorf, Germany; 3 Department of Cellular and Molecular Pharmacology, University of California San Francisco, San Francisco, California, United States of America; 4 MRC Human Genetics Unit, Western General Hospital, Edinburgh, Scotland, United Kingdom; University of Pittsburgh, United States of America

## Abstract

Cells are regularly exposed to stress conditions that may lead to protein misfolding. To cope with this challenge, molecular chaperones selectively target structurally perturbed proteins for degradation via the ubiquitin-proteasome pathway. In mammals the co-chaperone BAG-1 plays an important role in this system. BAG-1 has two orthologues, Bag101 and Bag102, in the fission yeast *Schizosaccharomyces pombe*. We show that both Bag101 and Bag102 interact with 26S proteasomes and Hsp70. By epistasis mapping we identify a mutant in the conserved kinetochore component Spc7 (Spc105/Blinkin) as a target for a quality control system that also involves, Hsp70, Bag102, the 26S proteasome, Ubc4 and the ubiquitin-ligases Ubr11 and San1. Accordingly, chromosome missegregation of *spc7* mutant strains is alleviated by mutation of components in this pathway. In addition, we isolated a dominant negative version of the deubiquitylating enzyme, Ubp3, as a suppressor of the *spc7-23* phenotype, suggesting that the proteasome-associated Ubp3 is required for this degradation system. Finally, our data suggest that the identified pathway is also involved in quality control of other kinetochore components and therefore likely to be a common degradation mechanism to ensure nuclear protein homeostasis and genome integrity.

## Author Summary

The accumulation of misfolded proteins represents a considerable threat to the health of individual cells and has been linked to severe diseases, including cancer and neurodegenerative disorders. To cope with this threat, especially under stress conditions, cells have evolved efficient quality control mechanisms. In general, these rely on molecular chaperones to either seize and refold misfolded proteins, or target them for degradation via the ubiquitin-proteasome system. At present, our understanding of what determines whether a chaperone commits to a folding or a degradation mode is limited. However, studies suggest that association with certain regulatory co-chaperones contributes to this process. Here, we show that certain BAG-1-type co-chaperones function in quality control by targeting misfolded kinetochore components for proteolysis. The presented genetic and biochemical data show that specific ubiquitin conjugating enzymes and ubiquitin-protein ligases maintain nuclear protein homeostasis and are required for upholding genome integrity.

## Introduction

Various conditions may cause partial denaturation of cell proteins. Such proteins are either shielded from aggregation and refolded to the native state by molecular chaperones [1] or they are targeted for degradation via the ubiquitin-proteasome system (UPS) [2] [3] [4] [5] [6] [7].

Faults in this system may lead to a build-up of toxic protein species which may cause diseases, including neurodegenerative disorders such as Parkinson's and Alzheimer's disease. Conversely, chaperone-assisted degradation of proteins that are structurally perturbed, but still functional, has also been linked to disease, as in cystic fibrosis [8].

At present, our understanding of what determines whether a chaperone commits to a folding or a degradation mode is limited. However, studies suggest that association with certain regulatory co-chaperones contributes to this process [9] [3]. The human BAG-1 anti-apoptotic protein is one such cofactor for Hsp70-type chaperones [10]. The BAG-1 co-chaperone functions as a nucleotide exchange factor for the chaperone and triggers the release of bound clients [10] [11] [12]. This substrate release is triggered by the BAG domain in BAG-1, which binds to the ATPase domain of Hsp70 [10] [13] [11]. Thus, when BAG-1 associates with the 26S proteasome via its N-terminal ubiquitin-like (UBL) domain, chaperone-bound clients are released and degraded [10] [13] [14] [11]. For efficient degradation, chaperone clients must first be ubiquitylated. In mammalian cells this is accomplished by the E2 ubiquitin conjugating enzyme, Ubc4, and the E3 ubiquitin-protein ligase, CHIP [14] [15] [16]. Importantly, chaperone-assisted degradation of certain clients is not affected in CHIP-deficient mammalian cells [17], suggesting that other, unidentified, ubiquitin ligases maintain chaperone-assisted degradation in the absence of CHIP [18]. Since the human genome encodes hundreds of E3s, identifying these ubiquitin ligases might be more straightforward in a simpler and genetically more tractable model organism. A database search for orthologues of human BAG-1 in the fission yeast, *Schizosaccharomyces pombe*, revealed two BAG-1 homologues, Bag101 and Bag102. Here, we show that Bag101 and Bag102 localize to the cytosol and nuclear envelope, respectively. Both proteins associate with the 26S proteasome and Hsp70 chaperones. By systematic high-throughput genetic screening, we identified a mutant version of the Spc7 protein, Spc7-23, as a substrate for chaperone-assisted degradation in *S. pombe*. Spc7 belongs to the Spc105-KNL-1-Blinkin-Spc7 family, which comprises an essential component of the kinetochore, a macromolecular structure that mediates bipolar attachment of chromatids to spindle microtubules and controls mitosis [19]. Accordingly, the *spc7-23* mutant displays severe defects in chromosome segregation at the restrictive temperature [20]. In a *bag102*Δ background, the temperature-sensitive growth defect of the *spc7-23* mutant is alleviated and equal DNA segregation partially restored. We propose a model where Hsp70 monitors the quality of kinetochore subunits and via the E3 ubiquitin-protein ligases, Ubr11 and San1, and co-chaperone Bag102 targets kinetochore components for degradation. Importantly, the degradation also relies on the proteasome-associated deubiquitylating enzyme, Ubp3. Finally, we present evidence that this pathway also functions in the degradation of other mutant kinetochore components, including Mal2-1 and Mis6-302, suggesting that this pathway is involved in a general nuclear protein quality control system, required for correction transmission of genetic information.

## Results

### The fission yeast BAG domain proteins are homologues of human BAG-1

A database search for orthologues of human BAG-1 revealed that the fission yeast *S. pombe* encodes two UBL/BAG domain proteins, Bag101 and Bag102. The budding yeast, *Saccharomyces cerevisiae*, on the other hand, encodes only one BAG domain protein, Snl1. However, Snl1 does not contain any UBL domain (Fig. S1).

**Figure 1:**
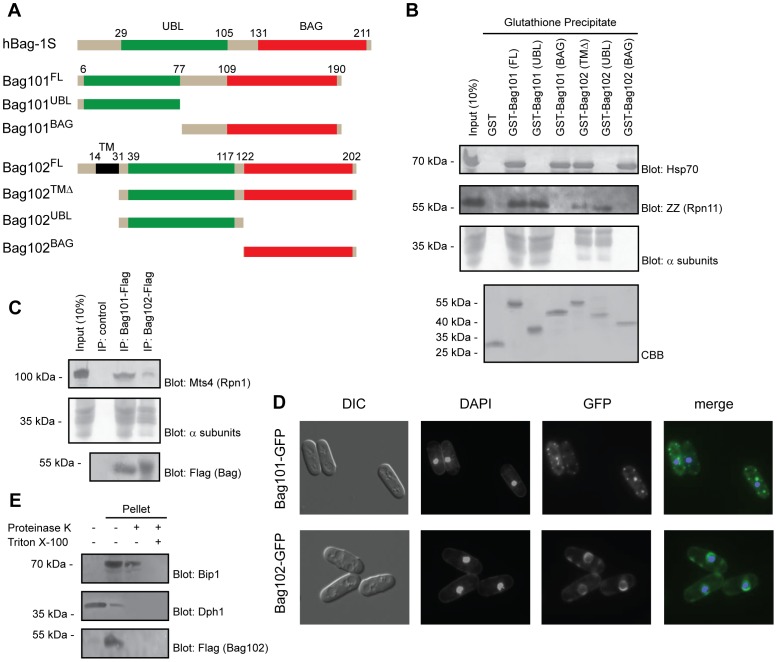
Bag101 and Bag102 interact with 26S proteasomes and Hsp70. (A) Domain organization (shown to scale) of human BAG-1S and the *S. pombe* homologs Bag101 and Bag102. The truncations used for the precipitation experiments are shown. (B) The indicated GST fusion proteins were used in pull down experiments with extract from *S. pombe* cells expressing ZZ-tagged Rpn11. The precipitated material was analyzed by SDS-PAGE and blotting for Hsp70 (upper panel), the ZZ-tagged 26S proteasome subunit Rpn11, and 20S particle α subunits (middle panels). Equal loading was checked by staining with Coomassie Brilliant Blue (CBB) (lower panel). (C) Immunoprecipitates from wild type *S. pombe* cells expressing Flag-tagged Bag101 and Bag102 were resolved by SDS-PAGE and analyzed by blotting, using antibodies to the proteasome subunit Mts4/Rpn1, and Flag. (D) Differential interference contrast (DIC) and fluorescence micrographs of wild type *S. pombe* transformed to express GFP-tagged Bag101 and Bag102. DAPI staining was used to mark the nucleus. (E) Lysates from *S. pombe* cells transformed to express Flag- and GFP-tagged Bag102 were separated into a soluble fraction and pellet. The pellet fraction was then treated with proteinase K and Triton X-100 as indicated, before the samples were analyzed by SDS-PAGE and blotting. Dph1 served as a control for a soluble protein. Bip1 served as a control for an ER luminal protein.

Like human BAG-1, the fission yeast BAG proteins both contain an N-terminal UBL domain and a C-terminal BAG domain (Fig. 1A). Overall, the fission yeast proteins show about 20% sequence identity and 45% sequence homology to the small (hBAG-1S) isoform of human BAG-1 (Fig. S1).

### Bag101 and Bag102 interact with Hsp70-type chaperones

In humans, BAG domain proteins were found to associate with Hsp70 chaperones via their BAG domains [9]. To test whether this was also the case for the *S. pombe* orthologues, the full length and truncated GST fusion proteins were produced in *E. coli* (Fig. 1A) and immobilized to glutathione Sepharose. Since Bag102 is predicted to contain a single transmembrane (TM) domain near the N-terminus, we deleted the first 31 amino acids to circumvent the likely folding issues of a recombinant transmembrane protein, expressed in bacteria. The immobilized proteins were incubated with cell extracts from yeast expressing protein A ZZ-tagged Rpn11. The precipitated material was probed for the presence of Hsp70 using antibodies to Hsp70. Both GST-Bag101 and GST-Bag102 efficiently precipitated Hsp70 from the extracts (Fig. 1B). The *S. pombe* genome encodes five highly similar nuclear/cytosolic Hsp70 type chaperones. Since the Hsp70 antibody used here does not discriminate between these proteins, it is not possible to determine if Bag101 and Bag102 preferentially associate with specific Hsp70s. The use of truncated versions of Bag101 and Bag102 revealed that the interaction with Hsp70 is mediated by the C-terminal BAG domain (Fig. 1B). These data imply that Bag101 and Bag102 are similar to human BAG-1 in their BAG-dependent association with Hsp70.

### Bag101 and Bag102 associate with the 26S proteasome

Previously, several UBL domain proteins, including mammalian BAG-1, have been found to interact with 26S proteasomes [21] [13] [22] [23]. To map the interaction between the fission yeast UBL/BAG domain proteins and the 26S proteasome, the pull down samples (Fig. 1B) were also probed for the presence of 26S proteasomes using antibodies to the Rpn11 ZZ-tag and the 20S particle α subunits. In accordance with our expectations, full length Bag101 and Bag102 precipitated 26S proteasomes from the extracts (Fig. 1B) and the N-terminal UBL domains were necessary and sufficient to mediate the interaction (Fig. 1B). However, the proteasome binding of Bag102 appeared reduced compared to Bag101 (Fig. 1B).

To test if the UBL/BAG proteins also associate with 26S proteasomes *in vivo*, we performed co-immunoprecipitation experiments from cells expressing C-terminally Flag-tagged Bag101 and Bag102. We found that both Bag101 and Bag102 co-precipitated with 26S proteasomes (Fig. 1C). Together, these data imply that Bag101 and Bag102 associate with 26S proteasomes in a UBL domain dependent manner.

### Subcellular localization of Bag101 and Bag102

Next we examined the subcellular localization of Bag101- and Bag102-GFP fusion proteins. Bag101 was present throughout the cytosol and nucleus, but clearly enriched in several cytosolic spots (Fig. 1D). Bag102, on the other hand, localized to the nuclear/ER membrane (Fig. 1D), similar to 26S proteasomes [24]. Thus, one would predict that the Bag102 protein functions on the cytosolic and nuclear side of the nuclear/ER membrane, and not in the ER lumen. Accordingly, the Bag102 UBL and BAG domains should face the cytosol and nucleus. To test this prediction, microsomes were isolated from cells expressing Bag102 with a C-terminal Flag epitope and analyzed by blotting. In accordance with Bag102 containing a transmembrane domain, all Bag102 was associated with the microsome pellet (Fig. 1E). The membrane fraction only contained trace amounts of the soluble control protein Dph1 (Fig. 1E). Upon treating the microsomes with proteinase K, the ER luminal protein Bip1 was not affected, suggesting that the isolated microsomes were intact (Fig. 1E). However, the signal from the C-terminal Flag epitope on Bag102 was completely lost (Fig. 1E), so we conclude that Bag102 is a nuclear membrane protein that is oriented with the C-terminus facing the cytosol and/or nucleus.

### Epistatic miniarray profiling identifies Spc7-23 as a substrate for chaperone-assisted degradation

To further characterize the functions of Bag101 and Bag102 *in vivo*, *bag101-* and *bag102*-null mutants were constructed by PCR mutagenesis. Curiously, neither the *bag101*Δ nor the *bag102*Δ strain displayed any obvious growth phenotypes at 25°C or 37°C (Fig. S2). A *bag101*Δ*bag102*Δ double mutant also appeared as wild type (Fig. S2). Therefore, we performed an epistatic miniarray profiling (E-MAP) screen [25] on *bag101*Δ and *bag102*Δ mutants. By robotics, the *bag101*Δ mutant was systematically crossed to a collection of 393 other *S. pombe* mutants, while the *bag102*Δ strain was crossed to 540 mutants. This collection of mutants contained primarily verified null mutants where the coding sequence had been replaced with a nourseothricin resistance (clonNAT) cassette, but also included a few point mutants in essential genes with the clonNAT cassette inserted next to the mutant gene. The double mutant progeny was then scored for growth. Neither *bag101*Δ nor *bag102*Δ was found to display any strong negative genetic (synthetic sick/lethal) interactions with the mutants included in the screen. However, *bag101*Δ and *bag102*Δ did show some positive genetic interactions (synthetic rescue phenotypes) with certain mutants (Table 1 and Table 2. In particular, we noted that both *bag101*Δ and *bag102*Δ presented a synthetic rescue phenotype with a point mutant, *spc7-23* (Table 1 and Table 2), in the essential kinetochore component called Spc7 in *S. pombe* [26] [20], Spc105 in *S. cerevisiae*, and Blinkin in higher eukaryotes.

**Table 1:** Quantitative epistatic miniarray profiling for *bag101*Δ.

Score	*S. pombe*	*S. cerevisiae*	Function
−5.0	*ssp2*	*SNF1*	Ser/Thr protein kinase
−3.3	*SPAP27G11.07C*	*BUD32*	protein kinase
−3.1	*dsc3*	*YOR223W*	putative membrane protein
−3.0	*spf1*	*SPP1*	PHD finger protein
−3.0	*trt1*	*EST2*	telomerase reverse transcriptase
−2.9	*pac10*	*PAC10*	prefoldin subunit
−2.5	*SPBC660.05*	*WWM1*	conserved WW domain protein
+2.0	*rik1*	*-*	silencing protein, E3 subunit
+2.3	*klp2*	*KAR3*	kinesin-like protein
+6.5	*spc7-23*	*SPC105*	essential kinetochore protein

**Table 2:** Quantitative epistatic miniarray profiling for *bag102*Δ.

Score	*S. pombe*	*S. cerevisiae*	Function
−3.8	*sap114*	*PRP21*	splicing factor
−3.5	*raf1*	*-*	Rik1-associated factor, silencing
−3.4	*SPBC1348.10c*	*PLB*	phospholipase B
−2.9	*fin1*	*KIN3*	serine/threonine protein kinase
−2.8	*raf2*	*-*	Rik1-associated factor
−2.7	*prz1*	*CRZ1*	transcription factor
−2.6	*SPAC17A5.09c*	*GLC8*	phosphatase regulatory subunit
−2.6	*atg6*	*VPS30*	beclin family protein
+2.1	*SPCC162.11c*	*URK1*	uridine kinase
+2.2	*SPAC24B11.07C*	*PAM1,SVL3*	oxidoreductase (predicted)
+2.4	*rpb7*	*RPB7*	RNA polymerase II subunit
+2.5	*SPCC1919.03C*	*GAL83, SIP2*	AMP-activated kinase subunit
+2.5	*SPBP8B7.08C*	*PPM1*	Leu carboxyl methyltransferase
+3.2	*cdt2*	*-*	regulator of E3 activity
+4.8	*spc7-23*	*SPC105*	essential kinetochore protein

**Figure 2:**
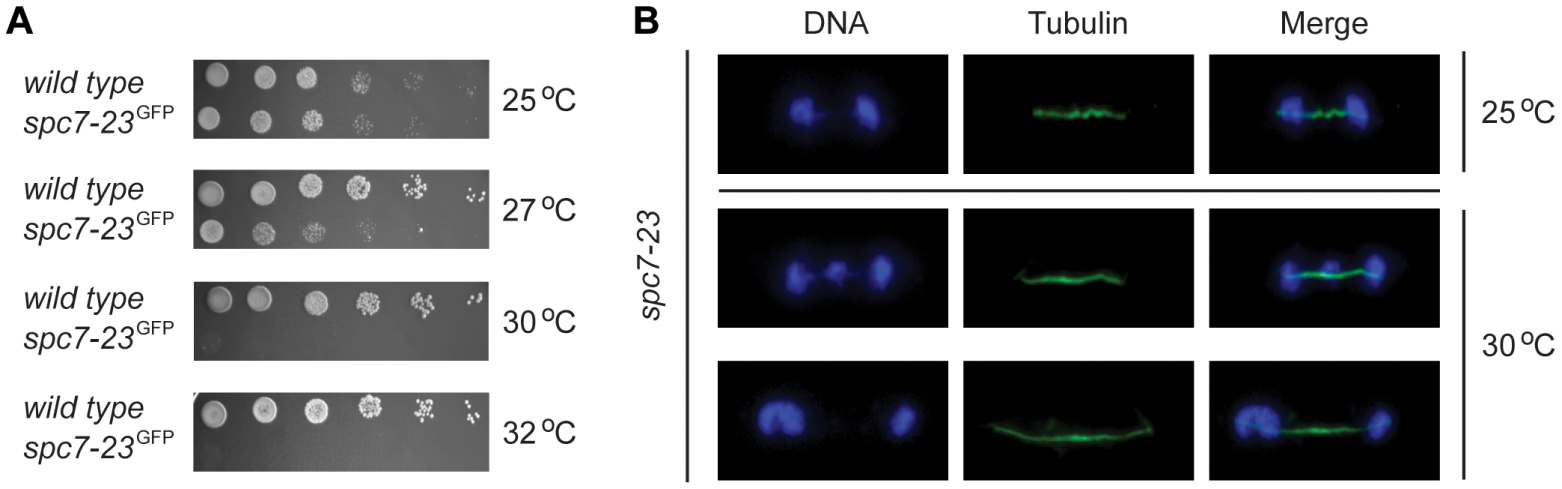
*spc7-23* cells are temperature sensitive and defective in DNA segregation. (A) The growth of wild type and *spc7-23* cells on solid media was compared at the indicated temperatures. (B) Cells carrying the mutant *spc7-23* mutation were incubated at 25°C or 30°C and stained to visualize DNA (blue) and tubulin (green). Note that the DNA segregation becomes unequal at the restrictive temperature.

**Figure 3:**
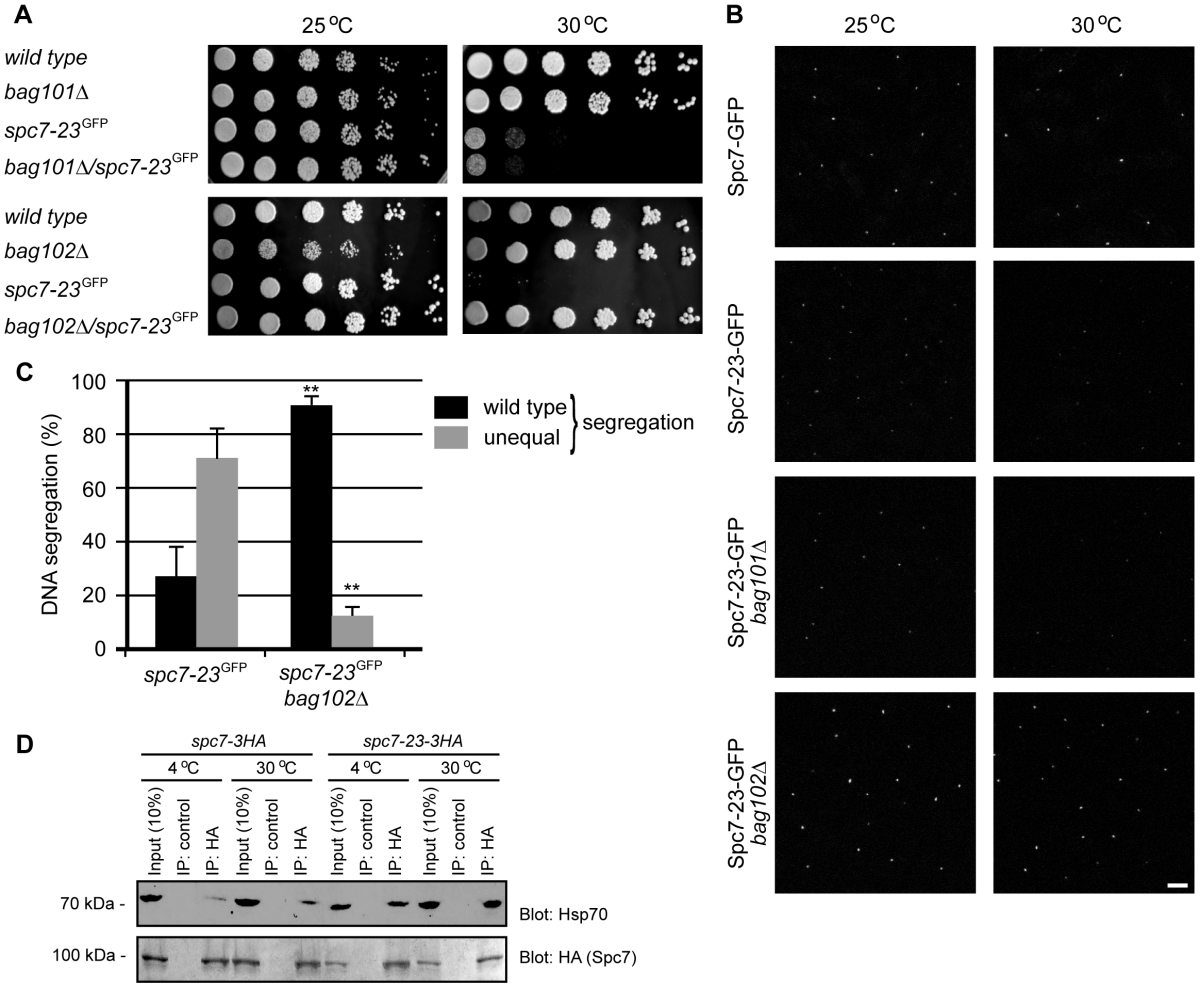
Spc7-23 levels are regulated by Bag102. (A) The growth on rich media of wild type, *bag101*Δ, *bag102*Δ, *spc7-23* and double mutants was compared at the indicated temperatures. (B) The abundance and kinetochore localization of the Spc7-23-GFP protein or, as a control, wild type Spc7-GFP, was analyzed in cells at 25°C (left panel) or at 30°C for 6 hours (right panel). Each fluorescent signal represents the Spc7-23-GFP kinetochore signal of an individual interphase cell as kinetochores are clustered at the spindle pole body during interphase. Note that at 30°C the Spc7-23-GFP signal is reduced in a wild type (*bag102* ^+^) or *bag101*Δ background. In a *bag102*Δ background the Spc7-23-GFP signal is increased at both temperatures. Scale bar: 5 µm. (C) Equal (wild type) or unequal DNA segregation was quantified in *spc7-23-gfp* and *spc7-23-gfp bag102*Δ cells at 30°C. ** p<0.01 (Welch test) for the *spc7-23-gfp bag102*Δ strain compared to the *spc7-23-gfp* strain. For *spc7-23-gfp* (n = 200) and *spc7-23-gfp bag102*Δ (n = 100) late anaphase cells. Note that wild type DNA segregation is re-established in the *spc7-23bag102*Δ double mutant. (D) Spc7-3HA or Spc7-23-3HA was precipitated using antibodies to HA or control antibodies of the same isotype against an irrelevant protein (α2-macroglobulin) at either 4°C or 30°C. The precipitated material was resolved by SDS-PAGE and analyzed by blotting for the presence of Hsp70 (upper panel). Blotting to HA (Spc7 and Spc7-23) served as a loading control (lower panel). Note that Spc7-23 interacts with Hsp70, especially at 30°C.

We decided to have a closer look at the *spc7-23* mutant. In agreement with what has been observed before [20], we found that the *spc7-23* allele is temperature sensitive for growth (Fig. 2A) and displays a severe DNA segregation defect at the restrictive temperature (Fig. 2B). Rescue of the Spc7 mutant by mutations in *bag101* and *bag102* (Tables 1 and 2), and the association of BAG proteins with the 26S proteasome suggest that when the BAG proteins are lacking, Spc7-23 degradation is impaired, and growth therefore restored. To test this hypothesis, we first set out to verify the result of the E-MAP screen. We combined the *spc7-23* mutation with null mutants in *bag101* ^+^ and *bag102* ^+^. In the case of the *spc7-23bag101*Δ, we did not observe any synthetic rescue of the temperature sensitive phenotype (Fig. 3A), indicating that *bag101*Δ was a false positive in the E-MAP screen. However, growth of the *spc7-23bag102*Δ double mutant was completely restored at 30°C (Fig. 3A), and ectopic expression of the Bag102-GFP fusion protein reversed this effect (Fig. S3).

Using live cell imaging we determined if kinetochore targeting of Spc7-23-GFP was altered in a *bag102* deletion background. Spc7-GFP and Spc7-23-GFP can be seen as one single fluorescent signal per interphase cell grown at 25°C (Fig. 3B). However at the non-permissive temperature of 30°C the mutant Spc7-23-GFP protein is no longer kinetochore targeted. This abnormal phenotype was suppressed in a *bag102*Δ background (Fig. 3B, Fig. S4, Fig. S5).

We then asked if the DNA segregation defect of the *spc7-23* mutant was also suppressed in the *bag102*Δ background. At the restrictive temperature, the *spc7-23* single mutant displayed an uneven DNA distribution in approximately 70% of the mitotic cells (Fig. 3C), while in the *spc7-23bag102*Δ strain this was reduced to less than 15% (Fig. 3C).

If Spc7-23 is indeed a substrate for chaperone-assisted degradation, one would expect Spc7-23 to be associated with molecular chaperones such as Hsp70. To test this prediction, we immunoprecipitated Spc7 and Spc7-23 from *S. pombe* lysates at either 4°C or 30°C. As has been noted before [27] [20] [28], we found that Spc7 (and Spc7-23) migrated faster in SDS-PAGE gels than predicted from the calculated molecular weight (153 kDa). In general, the chaperone requirement of proteins is more stringent at higher temperatures. Accordingly, we observed more Spc7-23 associated with Hsp70 at 30°C than at 4°C (Fig. 3D). Also, the interaction between Spc7-23 and Hsp70 appeared much stronger than between wild type Spc7 and Hsp70 (Fig. 3D).

Together, these data support a model where Bag102, but not Bag101, is required for a nuclear chaperone-assisted degradation mechanism that ensures kinetochore integrity.

### Degradation of Spc7-23 depends on the 26S proteasome

**Figure 4:**
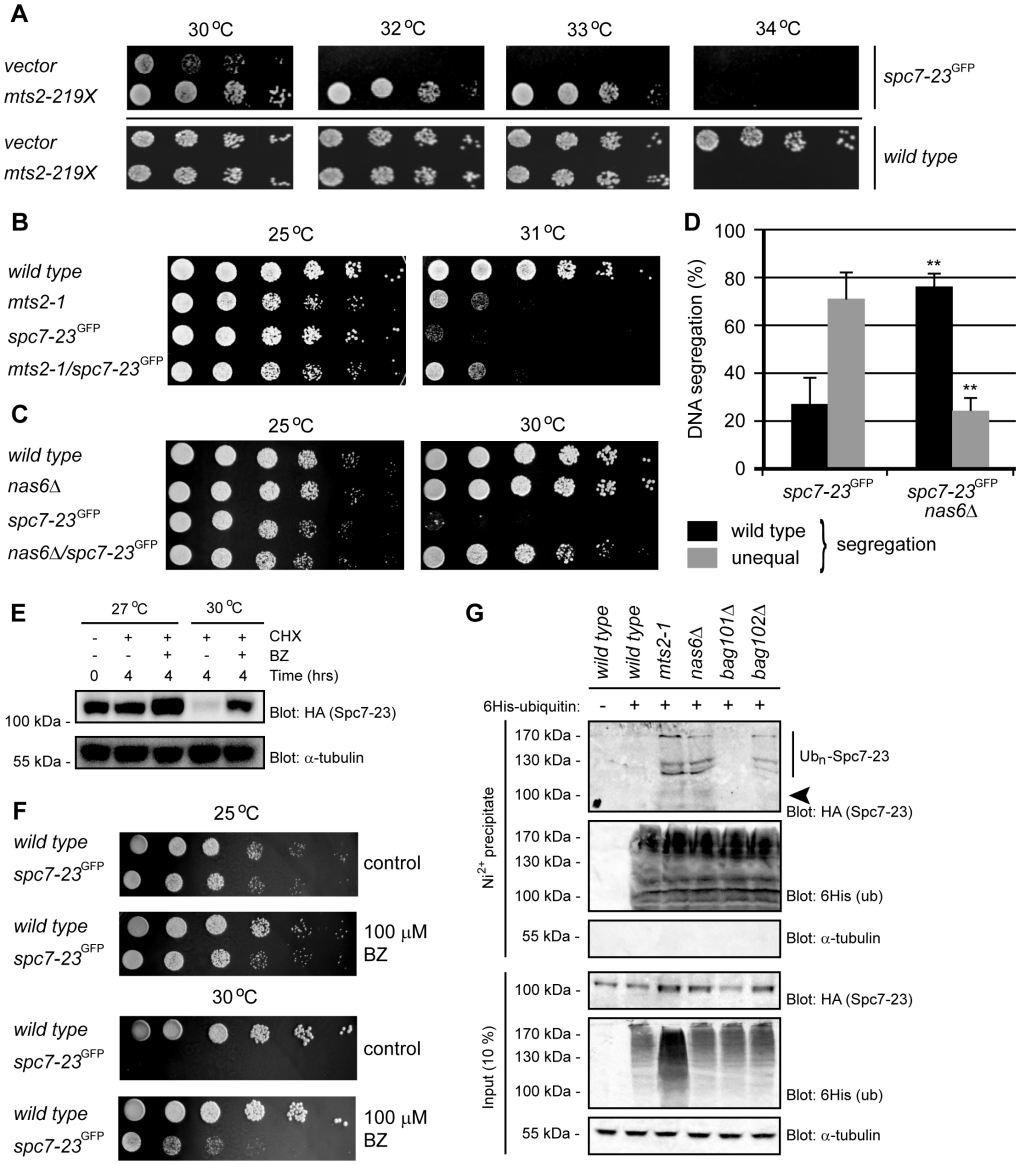
Spc7-23 is degraded via the ubiquitin-proteasome pathway. (A) The growth on solid media of wild type (lower panel) and *spc7-23* cells (upper panel) transformed with either a control vector (vector) or PstI digested genomic DNA construct encoding *mts2-219X* was compared at different temperatures. (B) The growth of wild type, *mts2-1*, *spc7-23* and the *mts2-1spc7-23* double mutant was compared at the indicated temperatures. (C) The growth on solid media of wild type, *nas6*Δ, *spc7-23* and the *nas6*Δ*spc7-23* double mutant was compared at the indicated temperatures. (D) Equal (wild type) or unequal DNA segregation was quantified in *spc7-23-gfp* and *spc7-23-gfp nas6*Δ cells at 30°C. ** p<0.01 (Welch test) for the *spc7-23-gfp nas6*Δ strain compared to the *spc7-23-gfp* strain. For *spc7-23-gfp* (n = 200) and *spc7-23-gfp nas6*Δ (n = 100) late anaphase cells. Note that wild type DNA segregation is re-established in the *spc7-23nas6*Δ double mutant. (E) The amount of Spc7-23 protein was followed in cultures at 27°C and 30°C where protein synthesis was inhibited with 100 µg/mL cycloheximide (CHX) for 4 hours. To some cultures 1 mM of the proteasome inhibitor Bortezomib (BZ) was also added. Equal loading was checked using antibodies to tubulin. (F) The growth on solid media of wild type and *spc7-23* cells was compared at the indicated temperatures in the absence (control) or presence of 100 µM BZ. (G) Strains with the indicated genetic backgrounds and transformed to express 6His-tagged ubiquitin were lysed and used for precipitation experiments with a Ni^2+^ resin in 8 M urea. The precipitated material was analyzed by blotting with antibodies to the HA-tag on Spc7-23 or to the 6His tag on ubiquitin. The arrowhead marks the position where non-ubiquitylated Spc7-23 migrates. Note that ubiquitylated Spc7-23 species are visible in proteasome and *bag102*Δ mutants.

Additional information on this Spc7 degradation pathway came from an extragenic suppressor screen of the *spc7-23* mutant phenotype [28]. One of the suppressing plasmids contained a genomic DNA fragment of chromosome II encoding a truncated version of the proteasome subunit Mts2/Rpt2, 5S rRNA, aspartate aminotransferase and a truncated version of the Mpr1 phosphotransferase. Digesting this plasmid with the PstI restriction enzyme generated a shorter construct encoding only the first 658 bp (219 aa) of *mts2* ^+^/*RPT2* and first 95 bp of the 5S rRNA. The temperature sensitive phenotype of the *spc7-23* strain transformed with this plasmid was repressed (Fig. 4A). Expression of the Mts2/Rpt2 truncation in a wild type background resulted in a temperature sensitive phenotype (Fig. 4A). Since the C-termini of proteasome Rpt subunits are critical for 26S proteasome assembly and function [29], expression of the truncated proteasome subunit Mts2/Rpt2 is likely to impede degradation of Spc7-23, thus restoring Spc7-23 protein levels and alleviating the growth and DNA segregation defects. Indeed, expression of the truncated Mts2 led to an increase in ubiquitin-protein conjugates (Fig. S6). To test the effect of proteasome mutants further, we crossed the temperature sensitive *mts2-1* mutant with the *spc7-23* strain and analyzed double mutant progeny for growth. At the permissive temperature, growth appeared normal. However, at the restrictive temperature, growth of the *spc7-23* mutant was severely compromised, while the *mts2-1spc7-23* double mutant appeared like the *mts2-1* single mutant (Fig. 4B). Furthermore, we combined the *spc7-23* mutant with a deletion in the proteasome assembly factor, *nas6* ^+^. Unlike the *mts2-1* strain, the *nas6*Δ strain is not overly temperature sensitive, and in this case we observed an almost complete restoration of growth in the double mutant at the restrictive temperature (Fig. 4C). We then asked if the DNA segregation defect of the *spc7-23* mutant was also suppressed in the *nas6*Δ background, like we observed in the *bag102*Δ background. Indeed, we found that the uneven DNA distribution of the *spc7-23* mutant was massively reduced upon loss of Nas6 (Fig. 4D).

Combined, these data suggest that the temperature sensitive and DNA segregation phenotypes of the *spc7-23* strain are, at least in part, caused by increased degradation of the Spc7-23 protein. To test this hypothesis, we followed the degradation of Spc7-23 in cultures, where protein synthesis was blocked with cycloheximide. Indeed, Spc7-23 protein appeared stable at the permissive temperature, but was rapidly degraded at the restrictive temperature (Fig. 4E). Moreover, this degradation was mediated by the UPS, since it was blocked by the proteasome inhibitor Bortezomib (BZ) (Fig. 4E). Accordingly, growth of the *spc7-23* strain at the restrictive temperature was restored on media containing sublethal amounts of Bortezomib (Fig. 4F). As has been shown before [30], we found that Bortezomib at 100 µM was not toxic to wild type fission yeast (Fig. 4F).

Together, these data suggest a model where, at the restrictive temperature, the mutant, but functional, Spc7-23 protein is detected by a quality control system and targeted for proteasomal degradation.

### Spc7-23 is ubiquitylated in proteasome and *bag102* mutants

To analyze this model further we decided to test the ubiquitylation status of Spc7-23. Cells were transformed with expression vectors for Spc7-23 and 6His-tagged ubiquitin. The ubiquitin was then precipitated under denaturing conditions (in 8 M urea), resolved by SDS-PAGE and analyzed by blotting. In agreement with the 26S proteasome being necessary for Spc7-23 degradation, Spc7-23 was ubiquitylated in both the *mts2-1* and *nas6*Δ backgrounds (Fig. 4G). No ubiquitylated Spc7-23 was detected in the *bag101*Δ cells (Fig. 4G). However, the *bag102*Δ strain did contain increased amounts of ubiquitylated Spc7-23 (Fig. 4G), suggesting that Bag102 functions at a step after ubiquitylation during Spc7-23 degradation.

### Degradation of Spc7-23 depends on the E2 Ubc4

**Figure 5:**
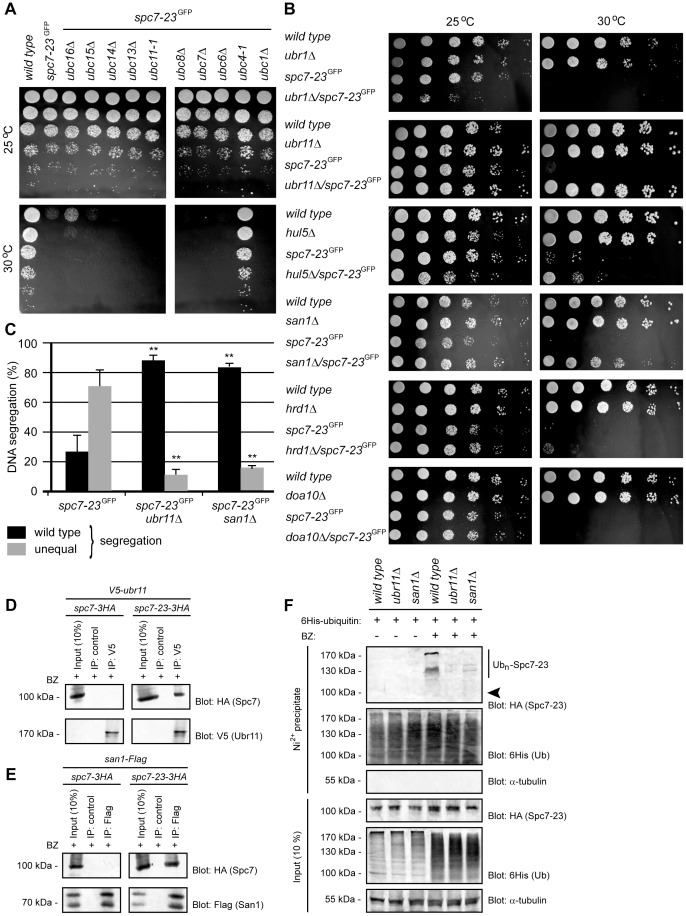
Spc7-23 degradation depends on Ubc4, Ubr11 and San1. (A) The growth of the indicated strains on solid media was compared at 25°C (upper panel) and 30°C (lower panel). (B) The growth of the indicated strains on solid media was compared at 25°C (left panel) and 30°C (right panel). (C) Equal (wild type) or unequal DNA segregation was quantified in *spc7-23-gfp ubr11*Δ and *spc7-23-gfp san1*Δ cells at 30°C. ** p<0.01 (Welch test) for the *spc7-23-gfp ubr11*Δ and *spc7-23-gfp san1*Δ strains compared to the *spc7-23-gfp* strain. For *spc7-23-gfp* (n = 200), *spc7-23-gfp ubr11*Δ (n = 100) and *spc7-23-gfp san1*Δ (n = 100) late anaphase cells. Note that wild type DNA segregation is re-established in the *spc7-23ubr11*Δ and *spc7-23san1*Δ double mutants. (D) V5-tagged Ubr11 was immunoprecipitated from cells treated with Bortezomib (BZ) using antibodies to V5 or control antibody. The precipitated material was analyzed by SDS-PAGE and blotting for V5 (Ubr11) and HA (Spc7). (E) Flag-tagged San1 was immunoprecipitated from cells treated with Bortezomib (BZ) using antibodies to Flag or control antibody. The precipitated material was analyzed by SDS-PAGE and blotting for Flag (San1) and HA (Spc7). (F) Strains with the indicated genetic backgrounds and transformed to express 6His-tagged ubiquitin were either not treated or treated with 1 mM of the proteasome inhibitor Bortezomib (BZ) overnight. 6His-tagged ubiquitin was precipitated with a Ni^2+^ resin in 8 M urea. The precipitated material was analyzed by SDS-PAGE and blotting with antibodies to the HA-tag on Spc7-23. The arrowhead marks the position where non-ubiquitylated Spc7-23 migrates. Note that ubiquitylated Spc7-23 species are less abundant in *ubr11*Δ and *san1*Δ mutants.

The data obtained so far suggest that kinetochore integrity is controlled by a chaperone-assisted degradation pathway involving the 26S proteasome, Hsp70 and the co-chaperone Bag102. In order to further characterize this degradation pathway, we aimed at identifying the E2 and E3 enzymes involved in the ubiquitylation reaction. Although *S. pombe* does not contain a CHIP orthologue, CHIP's cognate E2, Ubc4 [15], is present in this organism, making it a favored candidate for the degradation pathway. In order to identify the E2 involved in an unbiased fashion, we obtained deletion or conditional mutants in 11 of *S. pombe*'s ubiquitin-specific E2s and crossed them individually to the *spc7-23* strain to obtain double mutants. The *rhp6*Δ mutant, however, was very sick and we were unable to obtain a double mutant with *spc7-23*. Of the remaining 10 different *E2spc7-23* mutants, only the *ubc4-1spc7-23* mutant was viable at the restrictive temperature (Fig. 5A), suggesting that Ubc4 is indeed the sole or at least primary E2 ubiquitin conjugating enzyme involved in this degradation pathway.

### Degradation of Spc7-23 depends on the E3s Ubr11 and San1

In mammalian cells, chaperone-assisted degradation is generally thought to occur via the E3 ubiquitin-protein ligase, called CHIP. However, as mentioned, yeast cells do not encode any obvious CHIP orthologues. We therefore hypothesized that the relevant E3(s) involved in degradation of Spc7-23 are likely specific for misfolded proteins [18], and would localize to the nucleus or nuclear envelope and be induced by stress conditions such as high temperatures. These parameters led us to select 6 E3 candidates from available transcriptomics [31] and localization data [32]: Ubr1, Ubr11, Hul5, San1, Doa10 and Hrd1.

We then tested if null mutants in any of these E3s could suppress the temperature sensitive phenotype of the *spc7-23* strain. We found that growth at the restrictive temperature was fully restored in the *spc7-23ubr11*Δ and *spc7-23san1*Δ double mutants (Fig. 5B), but not in the *spc7-23ubr1*Δ, *spc7-23hrd1*Δ and *spc7-23doa10*Δ strains (Fig. 5B). The *spc7-23ubr1*Δ mutant appeared less fit than the single mutants at the permissive temperature. For the *spc7-23hul5*Δ strain, growth was only partially restored at the restrictive temperature (Fig. 5B). These results suggest that the primary E3s involved in Spc7-23 turnover are Ubr11 and San1. Accordingly, the uneven DNA segregation of the *spc7-23* strain was also strongly reduced in the *spc7-23ubr11*Δ and *spc7-23san1*Δ double mutants (Fig. 5C). To further analyze the genetic interactions between *ubr11*, *san1* and *spc7-23*, we isolated V5-tagged Ubr11 and Flag-tagged San1 by immunoprecipitation. We were unable to detect any V5-Ubr11 in extracts, but the protein was clearly enriched after precipitation (Fig. 5D). For both Ubr11 and San1 we found that Spc7-23, but not wild type Spc7, co-precipitated with the E3s when proteasome activity was inhibited with Bortezomib (Fig. 5D and Fig. 5E).

Finally, the increased ubiquitylation of Spc7-23 in response to Bortezomib was markedly decreased in the *ubr11*Δ strain, and also lowered in the *san1*Δ mutant (Fig. 5F), further corroborating that Spc7-23 is a target for Ubr11- and San1-catalyzed ubiquitylation and degradation.

### Degradation of Spc7-23 is regulated by the deubiquitylating enzyme Ubp3

**Figure 6:**
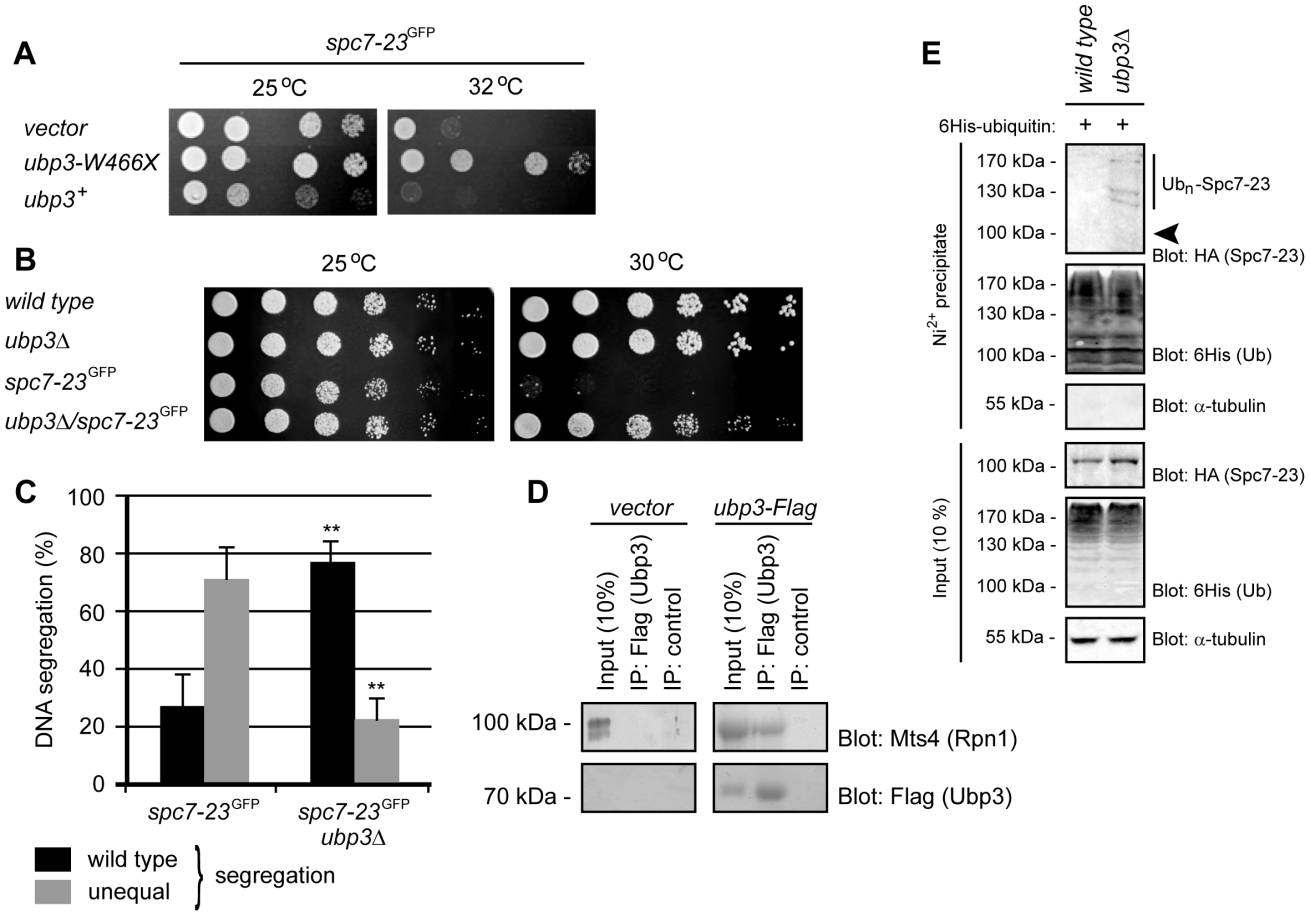
Spc7-23 degradation depends on the proteasome-associated DUB Ubp3. (A) The growth on solid media of *spc7-23* cells transformed with either a control plasmid (vector) or expression constructs for *ubp3* ^+^ and *ubp3-W466X* was compared at different temperatures. (B) The growth on solid media of wild type, *ubp3*Δ, *spc7-23* and the *ubp3*Δ*spc7-23* double mutant was compared at the indicated temperatures. (C) Equal (wild type) or unequal DNA segregation was quantified in *spc7-23-gfp* and *spc7-23-gfp ubp3*Δ cells at 30°C. ** p<0.01 (Welch test) for the *spc7-23-gfp ubp3*Δ strain compared to the *spc7-23-gfp* strain. For *spc7-23-gfp* (n = 200) and *spc7-23-gfp ubp3*Δ (n = 100) late anaphase cells. Note that wild type DNA segregation is re-established in the *spc7-23ubp3*Δ double mutant. (D) Cells transformed with vector (control) or Ubp3-Flag were used for immunoprecipitation experiments using antibodies to the Flag epitope. The precipitated material was analyzed by blotting for the proteasome subunit Mts4 (upper panel) or as a loading control Ubp3 (lower panel). (E) Wild type or *ubp3*Δ cells transformed to express 6His-tagged ubiquitin, were lysed and used for precipitation experiments with a Ni^2+^ resin in 8 M urea. The precipitated material was analyzed by blotting with antibodies to the HA-tag on Spc7-23 or the 6His tag on ubiquitin. The arrowhead marks the position where non-ubiquitylated Spc7-23 migrates.

In a suppressor analysis of the *spc7-23* mutant phenotype, carried out as described previously [28], we identified a truncated cDNA of the Ubp3 deubiquitylating enzyme (DUB). This truncated version of Ubp3 (Ubp3-W466X) lacks the last 47 residues of the enzyme, that includes part of the catalytic UCH domain, and therefore most likely functions as a dominant negative allele. To test this further, we transformed the *spc7-23* strain with either a control plasmid, or expression constructs for wild type full-length Ubp3 or the Ubp3-W466X truncation isolated in the screen. Indeed, expression of Ubp3-W466X restored growth at the restrictive temperature (Fig. 6A) while overexpression of wild type Ubp3 aggravated the growth defect (Fig. 6A). In agreement with this, deletion of *ubp3* ^+^ almost completely restored growth (Fig. 6B) and alleviated the DNA segregation defect (Fig. 6C) of the *spc7-23* strain at the non-permissive temperature. These data suggest that Ubp3 stimulates degradation of Spc7-23. In general, DUBs counter protein degradation. However, a few DUBs have been found to promote protein degradation. These are typically associated with the 26S proteasome, where release of the ubiquitin chain is required for efficient degradation [33] [34] [35]. Accordingly, we observed that 26S proteasomes co-precipitated with Ubp3 (Fig. 6D), as has been seen before [33], and that Spc7-23 was ubiquitylated in the *ubp3* null mutant (Fig. 6E).

### Other components follow the same degradation pathway

**Figure 7:**
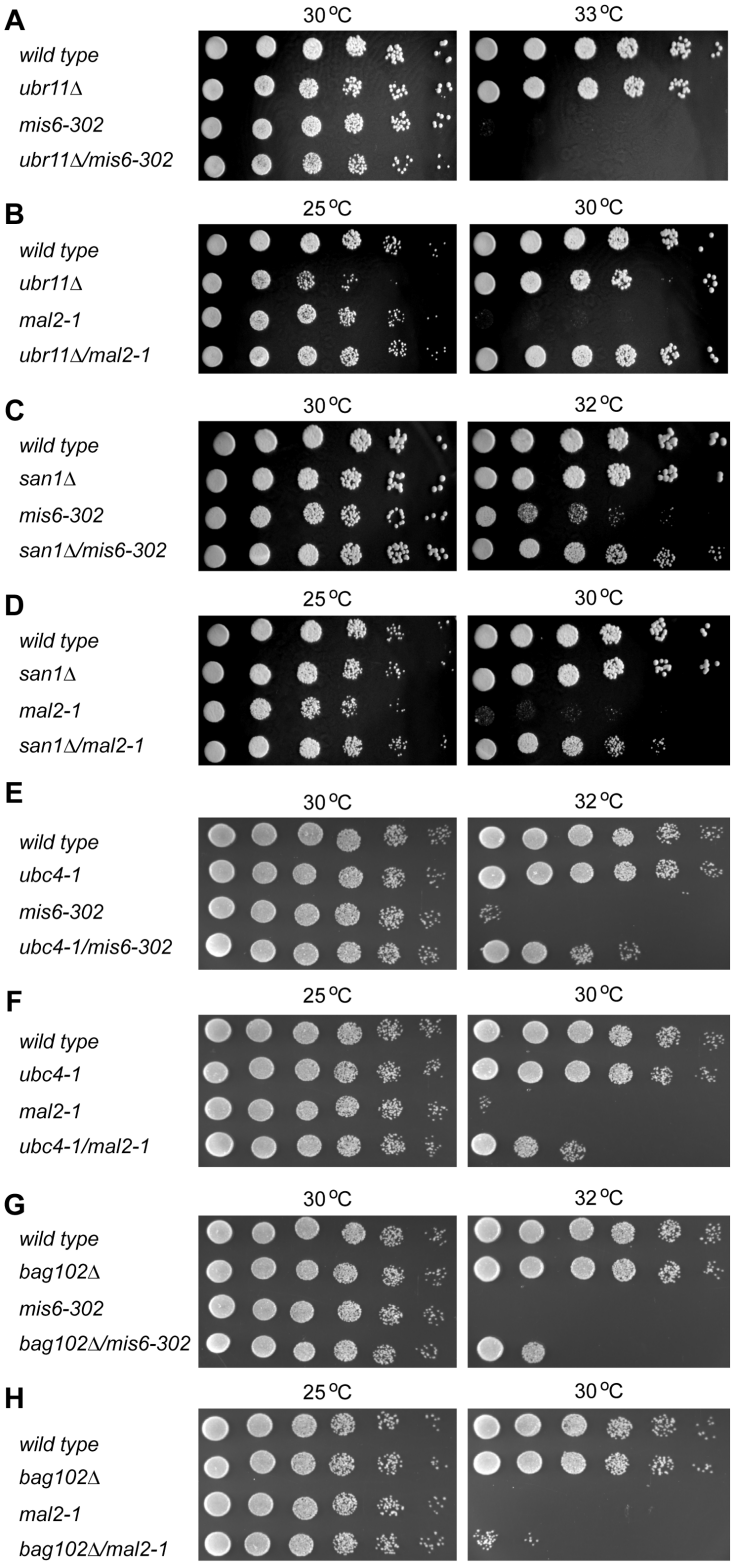
Other kinetochore mutants are also suppressed by *ubr11*Δ and *san1*Δ. (A) The growth on solid media of wild type, *ubr11*Δ, *mis6-302* and the *ubr11*Δ*mis6-302* double mutant was compared at the indicated temperatures. (B) The growth on solid media of wild type, *ubr11*Δ, *mal2-1* and the *ubr11*Δ*mal2-1* double mutant was compared at the indicated temperatures. (C) The growth on solid media of wild type, *san1*Δ, *mis6-302* and the *san1*Δ*mis6-302* double mutant was compared at the indicated temperatures. (D) The growth on solid media of wild type, *san1*Δ, *mal2-1* and the *san1*Δ*mal2-1* double mutant was compared at the indicated temperatures. (E) The growth on solid media of wild type, *ubc4-1*, *mis6-302* and the *ubc4-1mis6-302* double mutant was compared at the indicated temperatures. (F) The growth on solid media of wild type, *ubc4-1*, *mal2-1* and the *ubc4-1mal2-1* double mutant was compared at the indicated temperatures. (G) The growth on solid media of wild type, *bag102*Δ, *mis6-302* and the *bag102*Δ*mis6-302* double mutant was compared at the indicated temperatures. (H) The growth on solid media of wild type, *bag102*Δ, *mal2-1* and the *bag102*Δ*mal2-1* double mutant was compared at the indicated temperatures.

To further characterize this quality control system we decided to analyze if other kinetochore components follow the same degradation pathway as Spc7-23. To this end, we analyzed the temperature sensitive *mis6-302* and *mal2-1* mutant strains. Both Mis6 and Mal2 are part of the Sim4 kinetochore subcomplex [36] while Spc7 is part of the NMS complex. When these mutations were combined with *ubr11*Δ (Fig. 7A and Fig. 7B) viability was only restored at the restrictive temperature for the *mal2-1* strains. However, in case of *san1*Δ, viability at the restrictive temperature was restored for both the *mis6-302* and *mal2-1* strains (Fig. 7C and Fig. 7D). Mutation of *ubc4* also increased the viability of the *mis6-302* and *mal2-1* strains at the restrictive temperatures (Fig. 7E and Fig. 7F), as did deletion of *bag102*, although the effect was more modest than that observed for *spc7-23* (Fig. 7G and Fig. 7H).

This suggests that the degradation pathway, described here, is part of a more general control system that monitors the quality of several nuclear proteins, and especially kinetochore proteins.

## Discussion

Here, we describe a chaperone-assisted degradation pathway involved in nuclear protein quality control. Presumably this pathway targets a broad range of misfolded nuclear proteins, and not only kinetochore proteins. However, considering the extreme biological importance and complex nature of kinetochores, it is perhaps not surprising that a quality control system is involved in monitoring kinetochore integrity.

The precise transmission of chromosomes during cell division is a complex process that is highly regulated on several levels by kinetochores, which form a platform for checkpoint proteins, control microtubule plus-end dynamics and provide mechanical attachment sites for the spindle. Given the monumental tasks of a kinetochore, it is not surprising that these are complex structures of up to 100 different proteins that are organized in specific evolutionarily conserved subcomplexes. Although the composition of kinetochores is flexible to a certain degree, most kinetochore proteins are essential and distinct deviations from physiological levels of such proteins will lead to chromosome missegregation and aneuploidy [20] [37]. The folding, stoichiometry, and assembly of kinetochore components must therefore be tightly controlled. If the amount of a kinetochore component is insufficient or the protein is misfolded, complex formation may become blocked and chromosome segregation rendered defective. Accordingly, molecular chaperones play important roles in the biogenesis of functional kinetochores [38]. In general, proteins that do not pass inspection of chaperones are discarded via the UPS [18]. Data suggest that such quality control systems follow a better-safe-than-sorry principle, and are thus prone to target proteins that, albeit being structurally perturbed, are still functional. One example of this is in cystic fibrosis, where the degradation of certain mutant, but functional, versions of the chloride channel, CFTR, leads to a lowered amount of channel protein and manifestation of the disease [39]. Examples such as this have been instrumental for mapping of chaperone-assisted degradation pathways [18] and have recently been applied in linking the budding yeast E3, Ubr2, with regulating the stoichiometry of the budding yeast specific kinetochore component Dsn1 [40], presumably by a pathway related to the one described here. Intriguingly, some data suggest that not all kinetochore components follow the same degradation pathway. Previous studies focusing on the budding yeast kinetochore component Ndc10 have shown that an Ndc10 mutant that is only mildly structurally perturbed [41] is degraded via the ER-associated E3 ligase Doa10 [42]. The Ndc10 degron has also been defined, and is targeted by the Hsp70 chaperone Ssa1 [41].

Studies in mammalian cells have revealed that BAG-1 interacts with the 26S proteasome and Hsp70 [9] [3], thereby physically bridging the two major mechanisms for intracellular quality control, the degradation and refolding pathways. In essence, this also places BAG-1 at the centre of chaperone-assisted degradation. Hence, when chaperone clients cannot be refolded to their native conformation, E3 ligases, such as CHIP, first ubiquitylate them, while BAG-1 directs them for degradation by the UPS [9] [3]. Normally, substrate specificity is provided by the E3 ubiquitin ligase. However, the E3s involved in chaperone-assisted degradation appear to generally outsource substrate recognition to chaperones and co-chaperones [9] [3], thereby utilizing a pre-existing cellular system for recognition of misfolded proteins. Thus, in higher eukaryotes Hsp70s provide the substrate specificity by bringing the Hsp70 clients into proximity with the Hsp70-binding E3 ligase CHIP, before BAG-1 mediates the substrate release at the 26S proteasome [3] [5]. Exactly how this is accomplished for other E3s, such as Ubr11 is not know, but since Ubr11 interacts with Hsp70 [43], and the data presented here suggest that the role of UBL/BAG proteins is conserved, it is likely that the substrate selection and proteasomal delivery occurs by a similar mechanism. In regard to substrate specificity, San1, constitutes an exception from this rule, in that it, via intrinsically disordered areas, directly associates with a range of misfolded proteins [44]. However, certain San1 targets still associate with chaperones [45] and may require co-chaperones for proteasomal targeting [46].

Since targeted gene disruption of BAG-1 in mice leads to early embryonic lethality, due to massive cell death in the liver and severe defects in the differentiation and survival of neuronal cells [47], we were surprised to find that neither *bag101* nor *bag102* null mutants displayed any obvious phenotypes. However, yeast null mutants in other *bona fide* proteasome substrate targeting factors, such as Rhp23/Rad23 and Dph1/Dsk2, also do not display strong phenotypes, indicating a high level of redundancy in their *in vivo* functions [48].

Mammalian BAG-1 has been shown to interact with Hsp70-type chaperones via its BAG domain [10], and the structure of the BAG domain, bound to the ATPase domain of mammalian Hsp70, has been solved [11]. In *S. pombe*, we find that both Bag101 and Bag102 interact with Hsp70 via their BAG domain. Hence, biochemically, the Bag101 and Bag102 co-chaperones appear to exert similar functions. However, since only a mutant lacking Bag102 was able to suppress the phenotypes of the *spc7-23* strain and restore Spc7-23 protein levels, Bag101 and Bag102 appear to harbour separate and specific cellular functions. This is reflected by the difference in subcellular localization of Bag101 and Bag102. We found Bag102 at the nuclear membrane, presumably like 26S proteasomes [49], along the inside of the nuclear envelope, while Bag101 was mainly cytosolic.

**Figure 8:**
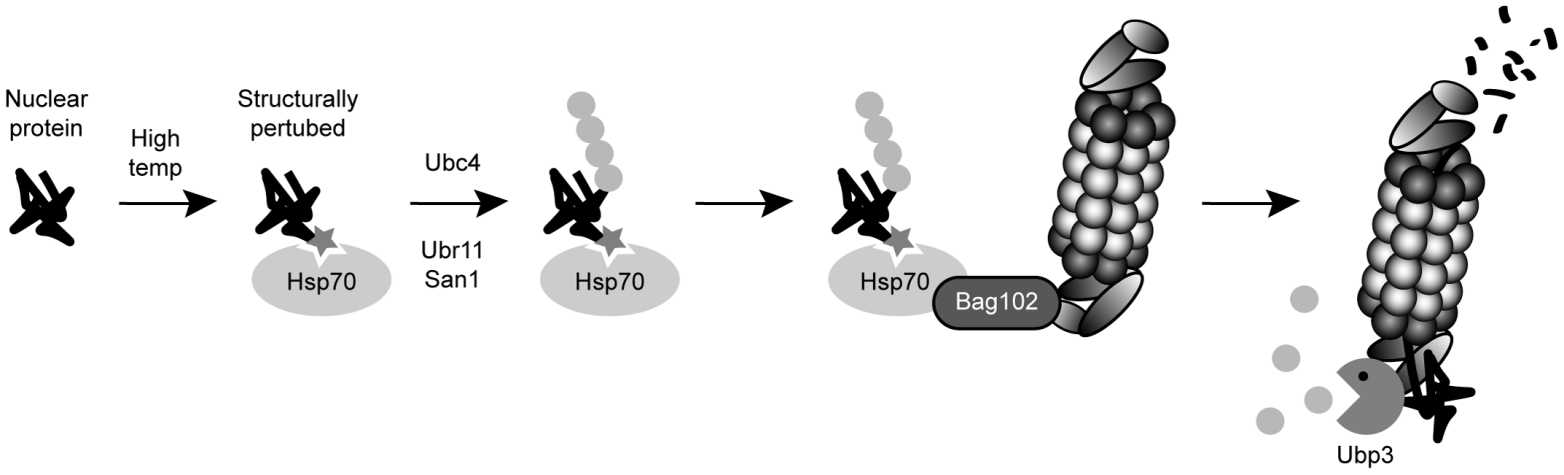
A chaperone-assisted degradation pathway for nuclear proteins. The data presented here are compatible with a model where a nuclear protein becomes structurally perturbed to a degree where it is still functional, but molecular chaperones detect it as being misfolded. The protein is then ubiquitylated by the E2 and E3 enzymes Ubc4, Ubr11 and San1, and directed to the 26S proteasome via Bag102. Finally, at the 26S proteasome, the protein is deubiquitylated by the DUB Ubp3 and degraded. Ubiquitin is shown as grey spheres. The structural perturbation is shown as a star.

We observed that the *spc7-23* phenotypes can also be alleviated by compromising other components of the chaperone-assisted degradation machinery. These data fit with a model where the Spc7-23 protein is slightly misfolded, but still functional, at the restrictive temperature. However, the surveillance system targets the protein for Ubr11- and San1-mediated ubiquitylation and proteasome-dependent degradation. This, in turn, leads to a reduced cellular level of the misfolded protein and manifestation of the phenotype (Fig. 8).

DUBs counter ubiquitylation and therefore play an important regulatory role in the UPS [50]. Our biochemical and genetic data and implicate the DUB, Ubp3, in promoting chaperone-assisted degradation. This effect is often tied to proteasome-associated DUBs that do not deubiquitylate the substrate until the substrate is committed to degradation [34] [33] [35]. Accordingly, Ubp3 was recently found to associate with 26S proteasomes and stimulate protein degradation in budding yeast [33]. Our data on *S. pombe* Ubp3 support this model. To our knowledge, Ubp3 is the first example of a DUB functioning in degradation of kinetochore proteins.

Remarkably, the degradation of chaperone clients proceeds normally in CHIP-deficient mammalian cells [17], suggesting that other ubiquitin ligases exert functions that overlap with CHIP during chaperone-assisted degradation. Though several candidates have been proposed [3], none of these are conserved to yeast. From the data presented here, we propose that Ubr11 functions in parallel with CHIP during chaperone-assisted degradation also in higher eukaryotes. Presumably due to its highly disordered structure and therefore poor sequence conservation, no San1 orthologue has yet been identified in higher eukaryotes. However, in case a San1 orthologue exists in these species, it is likely to functionally overlap with CHIP.

In conclusion, our data corroborate previous findings on mammalian BAG domain proteins [13] and reveal a so far unreported chaperone-assisted degradation pathway, involved in nuclear protein quality control and kinetochore integrity.

## Materials and Methods

### *S. pombe* strains and techniques

Fission yeast strains used in this study (Table S1) are derivatives of the wild type heterothallic strains *972h^−^* and *975h^+^*. Some strains were purchased from Bioneer [51]. Standard genetic methods and media were used and *S. pombe* transformations were performed using lithium acetate [52]. The PCR mutagenesis was performed as described [53].

### Plasmids

To generate Bag101 (SPBC16G5.11c) constructs, FL (full length), BAG domain (amino acids 78–190) and UBL domain (1–77) cDNAs were amplified from *S. pombe* genomic DNA and inserted into pDONR221 (Invitrogen) and pGEX-KG (GE Healthcare). Full length *bag102* ^+^ (SPBC530.03c), ΔTM (31–206), BAG (122–206) and UBL (31–121) cDNAs were also inserted into the pDONR221 and pGEX-KG vectors. For expression in *S. pombe*, the inserts from the pDONR221 vectors were transferred to the pDUAL vector system [54] using Gateway cloning technology (Invitrogen). Both *ubp3* variants were cloned via XhoI/NotI into the pJR2-3XL vector with the *nmt1* ^+^ promoter [55]. The HA-tagged Spc7-23 construct was obtained by QuikChange site directed mutagenesis (Stratagene) on pJR-XU41 plasmid encoding Spc7-3HA. The expression construct for 6His-ubiquitin has been described before [56]. The expression construct for San1 was kindly provided by Dr Makoto Kawamukai [57].

### Binding assays

The GST fusion proteins were expressed in *E. coli BL21 (DE3)* or *E. coli Rosetta (DE3)* and bound to glutathione Sepharose 4 beads (GE Healthcare) as described by the manufacturer. The protein/bead ratio was 1 mg/mL. Binding experiments were carried out using 20 µL of beads in 1 mL of 50% cleared (13000 g, 30 min.) extract from a strain expressing Pad1/Rpn11 with a Protein A ZZ-tag. The extract was prepared with glass beads in buffer A (25 mM Tris/HCl pH 7.5, 50 mM NaCl, 2 mM MgCl_2_, 5 mM ATP, 10% glycerol, 0.1% Triton X-100, 2 mM DTT, 1 mM PMSF and Complete protease inhibitors (Roche)). After overnight tumbling at 4°C, the beads were washed 4 times in 10 mL buffer A and resuspended in 30 µL SDS sample buffer. 20 µL of the samples were analyzed on 12% SDS gels and subjected to Western blotting.

Immunoprecipitations were performed from 50 mL cultures in mid exponential phase. Cells were lysed using glass beads in 1 volume of buffer B (25 mM Tris/HCl pH 7.5, 100 mM NaCl, 10% glycerol, 1 mM PMSF and Complete protease inhibitors (Roche)) and cleared by centrifugation (13000 g, 30 minutes). The soluble fraction was tumbled end-over-end for 4 hours at 4°C with 1 µL antibody to V5 (Invitrogen), Flag (Sigma) or HA (Sigma) and 20 µL protein G Sepharose (GE Healthcare). A monoclonal antibody of the same isotype but reacting with human α2-macroglobulin was used as a negative control [58]. The beads were then washed 4 times in 10 mL of buffer B and resuspended in 30 µL SDS sample buffer. 20 µL of the samples were separated on 12% SDS gels and subjected to Western blot analysis. All ubiquitin blots were boiled for 5 minutes prior to blocking to enhance immunoreactivity.

The antisera, used in Western blots, were affinity purified rabbit polyclonal anti-Mts4, mouse monoclonal MCP231 to 20S proteasome α subunits (Enzo), 5A5 mouse monoclonal anti-Hsp70 (Abcam), mouse monoclonal anti-Flag (Sigma), mouse monoclonal anti-V5 (Invitrogen), rabbit anti-ubiquitin (DAKO Cytomation), TAT1 mouse monoclonal anti-tubulin (Abcam) and rat anti-HA (Roche). Secondary antibodies were purchased from DAKO Cytomation.

### Ubiquitylation of Spc7-23

The ubiquitylation status of Spc7-23 was determined by precipitating 6His-ubiquitin under denaturing conditions, electrophoresis, and blotting for Spc7-23 as described previously [56].

### Growth assays

Growth assays on solid media were performed essentially as described [24]. Briefly, the *S. pombe* strains to be assayed were grown to an OD_600 nm_ of 0.4–0.8. The cells were then diluted in media to an OD_600 nm_ of exactly 0.40. Serial 5-fold dilutions of this culture were prepared before 5 µL of each dilution was spotted onto solid media plates (EMM2 for plasmid selection, otherwise YES) and incubated at the indicated temperature until colonies formed.

### Subcellular localization of Bag101 and Bag102

Cells transformed to express Bag101 or Bag102, containing a C-terminal GFP- and Flag-tag, were cultured in minimal media until mid exponential phase. The cells were fixed with 6% formaldehyde for 30 minutes and washed extensively with PBS. The fixed cells were mounted on cover slips using VectaShield Hard Set mounting media containing DAPI (Vector Laboratories) and analyzed in a fluorescence microscope (Zeiss AxioImager Z1) and CCD camera (Hamamatsu ORCA-ER).

Cells transformed to express Bag102 containing a C-terminal GFP- and Flag-tag were cultured in minimal media until late exponential phase. Then spheroplasts were prepared in buffer B (50 mM Tris/HCl pH 7.5, 2 mM MgCl_2_, 1 M sorbitol, 0.15% β-mercaptoethanol) containing 50 units/OD_600 nm_ Zymolase 100T (Saikagaku Co.) at 30°C for 1 hour. The cells were collected and lysed in buffer C (50 mM Tris/HCl pH 7.5, 1 mM EDTA, 0.2 M sorbitol) on ice by 20 strokes in a Dounce homogenizer. Unbroken cells were then removed by centrifugation at 500 g for 5 minutes. The supernatant was centrifuged at 13000 g for 15 minutes. The pellet (microsome fraction) was resuspended in buffer C, and then either mock incubated or incubated with 0.5 mg/ml proteinase K (Sigma) or proteinase K and 2% Triton X-100 for 2 hours at 30°C. Finally, the samples were analyzed by SDS-PAGE and blotting with antibodies to Flag (Sigma), Bip1 (generously provided by Dr John Armstrong) and Dph1 [48].

### Quantitative epistatic miniarray profiling (E-MAP)

The *bag101*Δ and *bag102*Δ mutants were screened for genetic interactions with a defined library of mutant nourseothricin (clonNAT)-resistant strains in the PEM2 background as described previously [25].

### Protein degradation assays

The degradation of the HA-tagged Spc7-23 protein was followed in cycloheximide-treated cultures by electrophoresis and blotting as described previously [59]. Antibodies to α-tubulin (Abcam) were used for the loading control. Bortezomib was purchased from LC Laboratories.

### DNA segregation

The appropriate strains were pregrown at 25°C, shifted to 30°C for 6 hours followed by fixation with formaldehyde. For immunofluorescence we used the anti-tubulin monoclonal TAT1 antibody followed by Alexa-Fluor 488-conjugated goat anti-mouse antibody (Invitrogen). DNA was stained with 4,6-diamidino-2-phenylindole (DAPI) (Sigma Aldrich). At least 2 experiments were carried out per strain.

### Live-cell imaging

For the data presented in Figure S5, strains were grown at the indicated temperatures, washed and stained with 10 µg/mL calcofluor white. The cell walls of cells of one strain were stained with 0.5 mg/mL TRITC-labeled lectin [60] prior to mixing with an equal amount of cells of the other strain.

For live-cell imaging a Zeiss spinning-disc confocal microscope with an AxioCam MRm camera, a temperature chamber and AxioVision software (Carl Zeiss Jena) were used at the Center for Advanced Imaging of the Heinrich Heine Universität Düsseldorf. The cells were pre-grown in minimal medium at 25°C, split and half of the culture shifted to 30°C for 6 hours. The cell samples were spotted on agarose pads as described [61]. All images in figure 3B were taken using the same conditions. Shown are maximum-intensity projections of 15 z slices with a distance of 0.5 µm using an argon laser (20% laser intensity, 400 ms exposure time). For figure S5 the following conditions were used: 50% 488 nm, 5% 561 nm, 3% 405 nm laser intensity.

Signal intensity of GFP fluorescence were measured in a 10-by-10 pixel square (with subtraction of the fluorescent background) in n = 30 cells per strain and temperature with ImageJ 1.44 software (NIH) as described in [28].

## Supporting Information

Figure S1 Sequence similarity of human BAG-1S, Bag101, Bag102 and Snl1. ClustalW alignments of human (*Hs*) BAG-1S, fission yeast (*Sp*) Bag101 (upper panel), Bag102 (middle panel) and budding yeast (*Sc*) Snl1 (lower panel). Identical and homologues residues are marked with (*) and (:), respectively. The domain organization is indicated by coloured bars.
(TIF)

Figure S2 No apparent phenotype of *bag101* and *bag102* null mutants. The growth on solid media of wild type, *bag101*Δ, *bag102*Δ and *bag101*Δ*bag102*Δ strains was compared at the indicated temperatures.
(TIF)

Figure S3 Expression of Bag102-GFP reverses the *bag102*Δ*spc7-23* phenotype. Growth comparison on solid media of wild type and *bag102*Δ*spc7-23* cells transformed with empty vector or the *bag102-GFP* ^+^ thiamine regulated NMT41 expression vector.
(TIF)

Figure S4 Quantification of Spc7-GFP and Spc7-23-GFP signals. The GFP signal intensities from Spc7-GFP or Spc7-23-GFP were quantified (from images as shown in figure 3C) for the indicated strains grown at 25°C (black) or 30°C (grey).
(TIF)

Figure S5 Live cell images of the indicated strains expressing Spc7-23-GFP. (A) The *spc7-23-gfp bag102Δ* cells were stained with TRITC-lectin before mixing them with an equal amount of *spc7-23-gfp* cells. (i) all cells were stained with calcofluor white; (ii) merged image showing caclofluor white and TRITC-lectin fluorescence; (iii) merged image showing TRITC-lectin fluorescence and Spc7-23-GFP signals; (iv) Spc7-23-GFP signals. Bar 10 µm. (B) The *spc7-23-gfp* cells were stained with TRITC-lectin before mixing them with an equal amount of *spc7-23-gfp bag102Δ* cells. (i) to (iv) as in (A).
(TIF)

Figure S6 Ubiquitin-protein conjugates are stabilized upon expression of *mts2-219X*. Total cell extracts were analyzed for ubiquitin-protein conjugates by SDS-PAGE blotting with antibodies to ubiquitin. Tubulin served as a loading control.
(TIF)

Table S1 Strains used. The fission yeast strains used in this study are listed.
(DOC)
