# Supplementary figures and images for: A Chaperone-Assisted Degradation Pathway Targets Kinetochore Proteins to Ensure Genome Stability
Source: PLoS Genet. 2014 Jan 30;10(1):e1004140. doi: 10.1371/journal.pgen.1004140 (PMC3907333; doi:10.1371/journal.pgen.1004140)

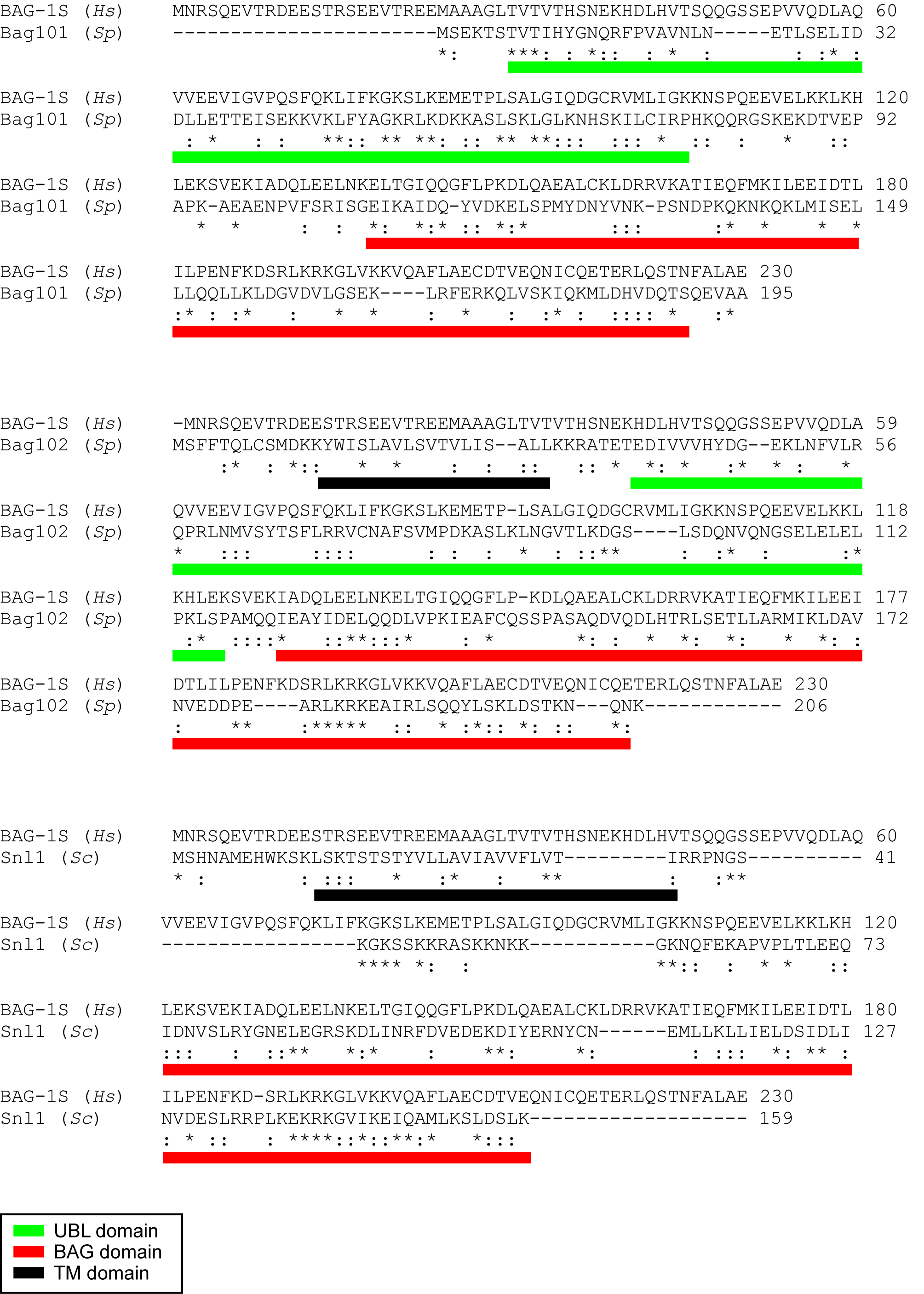

Supplement: Figure S1 — Sequence similarity of human BAG-1S, Bag101, Bag102 and Snl1. ClustalW alignments of human (Hs) BAG-1S, fission yeast (Sp) Bag101 (upper panel), Bag102 (middle panel) and budding yeast (Sc) Snl1 (lower panel). Identical and homologues residues are marked with (*) and (:), respectively. The domain organization is indicated by coloured bars. (TIF) [file pgen.1004140.s001.tif]

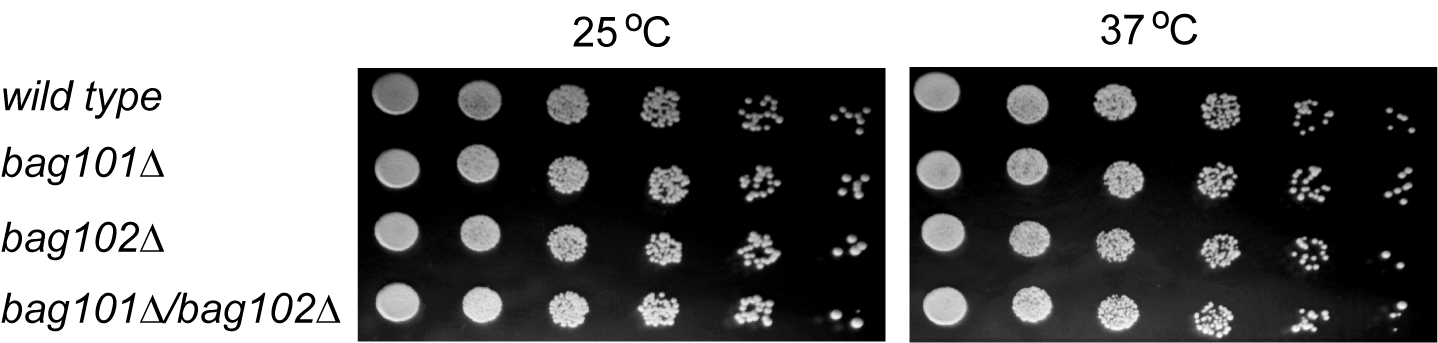

Supplement: Figure S2 — No apparent phenotype of bag101 and bag102 null mutants. The growth on solid media of wild type, bag101Δ, bag102Δ and bag101Δbag102Δ strains was compared at the indicated temperatures. (TIF) [file pgen.1004140.s002.tif]

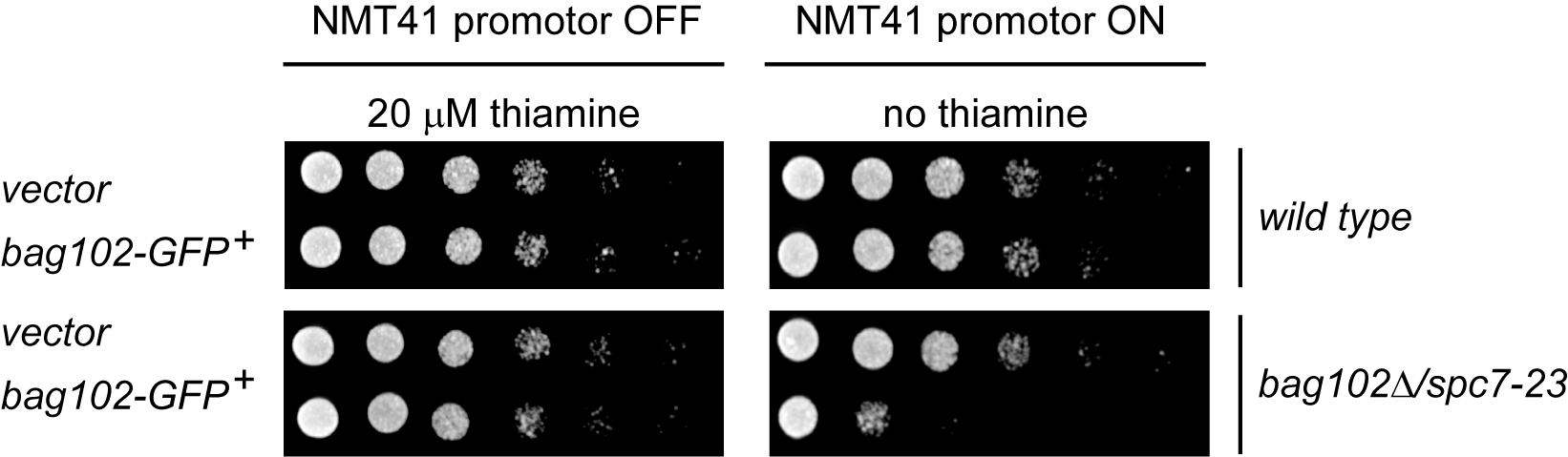

Supplement: Figure S3 — Expression of Bag102-GFP reverses the bag102Δspc7-23 phenotype. Growth comparison on solid media of wild type and bag102Δspc7-23 cells transformed with empty vector or the bag102-GFP + thiamine regulated NMT41 expression vector. (TIF) [file pgen.1004140.s003.tif]

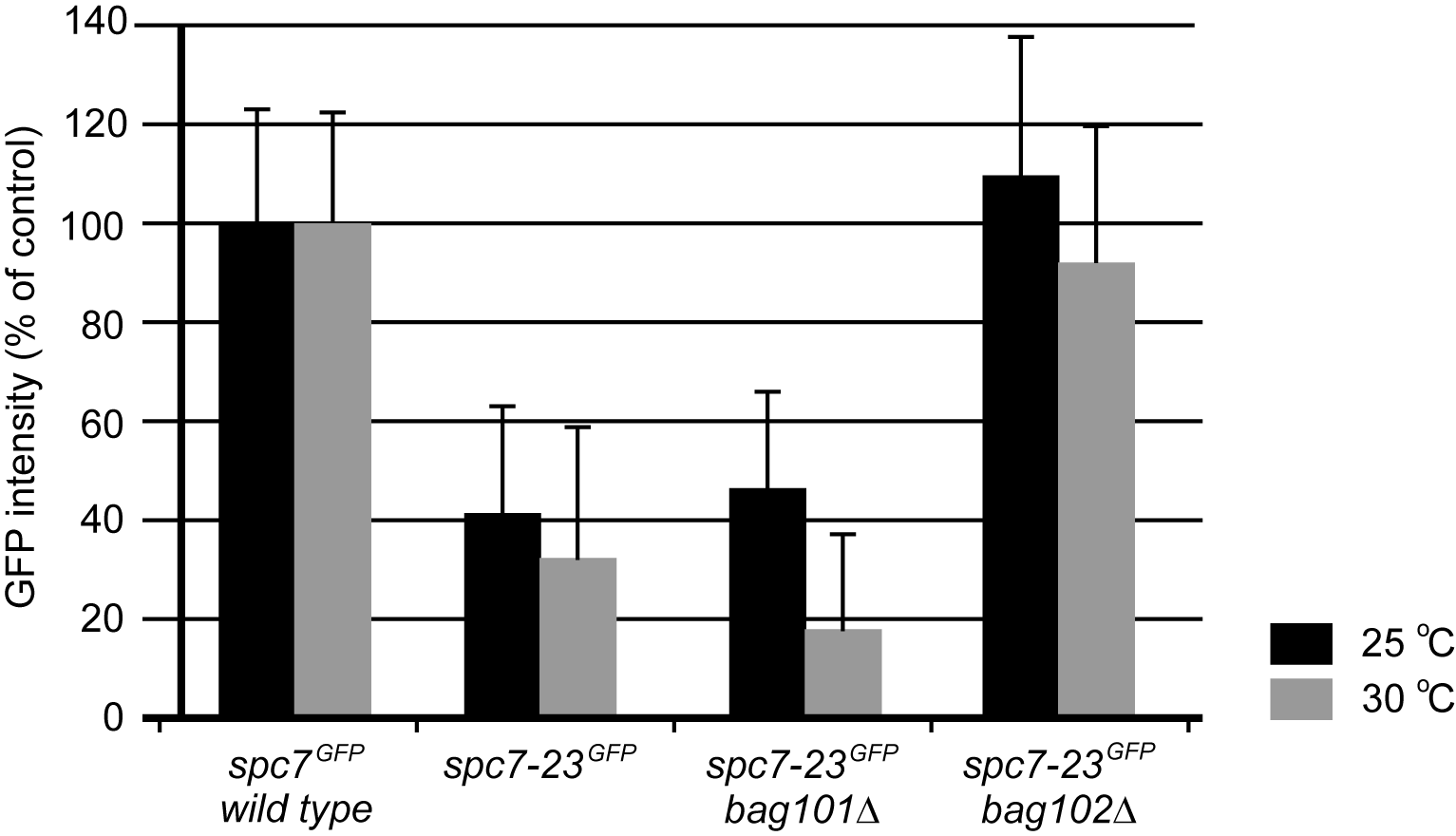

Supplement: Figure S4 — Quantification of Spc7-GFP and Spc7-23-GFP signals. The GFP signal intensities from Spc7-GFP or Spc7-23-GFP were quantified (from images as shown in figure 3C) for the indicated strains grown at 25°C (black) or 30°C (grey). (TIF) [file pgen.1004140.s004.tif]

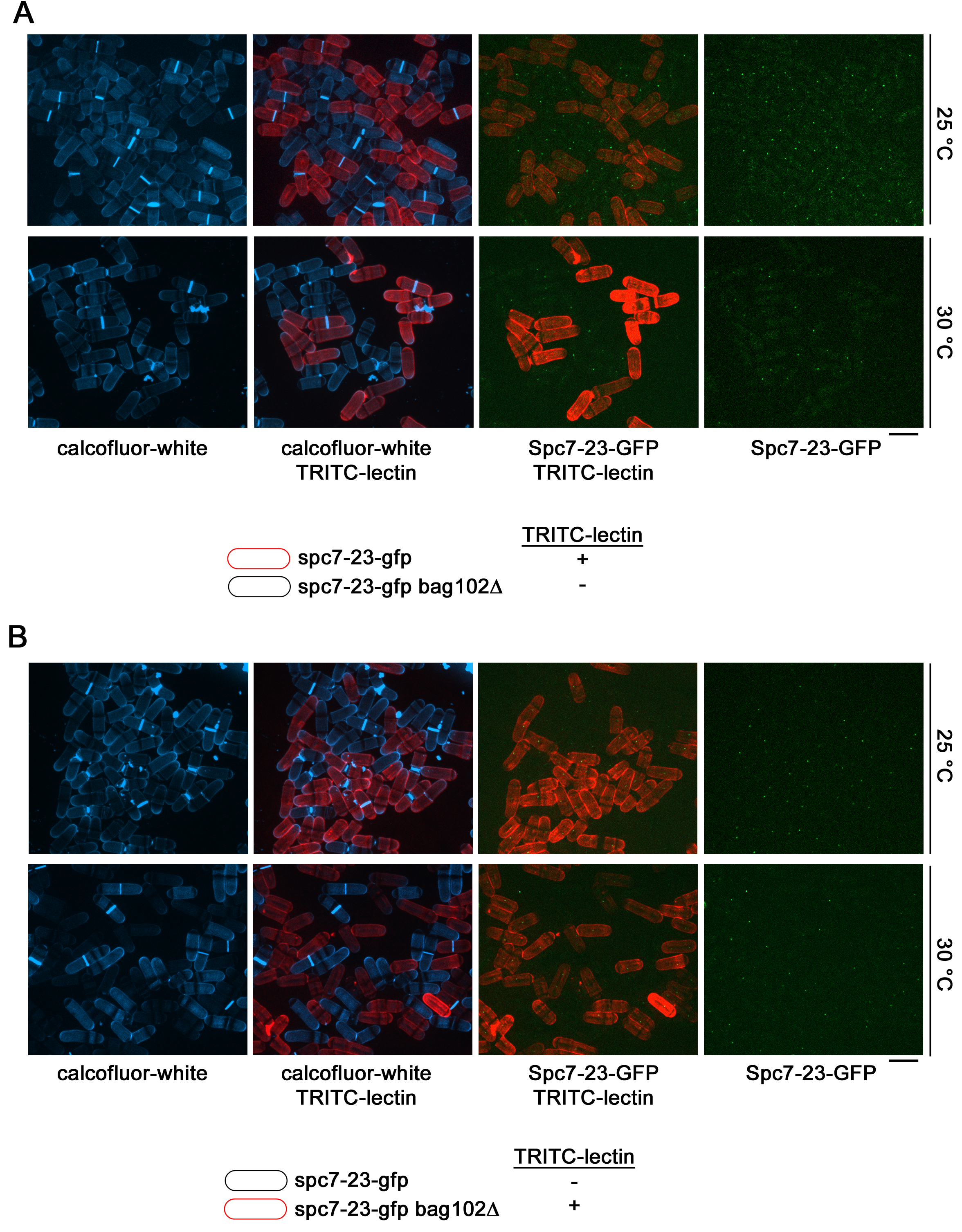

Supplement: Figure S5 — Live cell images of the indicated strains expressing Spc7-23-GFP. (A) The spc7-23-gfp bag102Δ cells were stained with TRITC-lectin before mixing them with an equal amount of spc7-23-gfp cells. (i) all cells were stained with calcofluor white; (ii) merged image showing caclofluor white and TRITC-lectin fluorescence; (iii) merged image showing TRITC-lectin fluorescence and Spc7-23-GFP signals; (iv) Spc7-23-GFP signals. Bar 10 µm. (B) The spc7-23-gfp cells were stained with TRITC-lectin before mixing them with an equal amount of spc7-23-gfp bag102Δ cells. (i) to (iv) as in (A). (TIF) [file pgen.1004140.s005.tif]

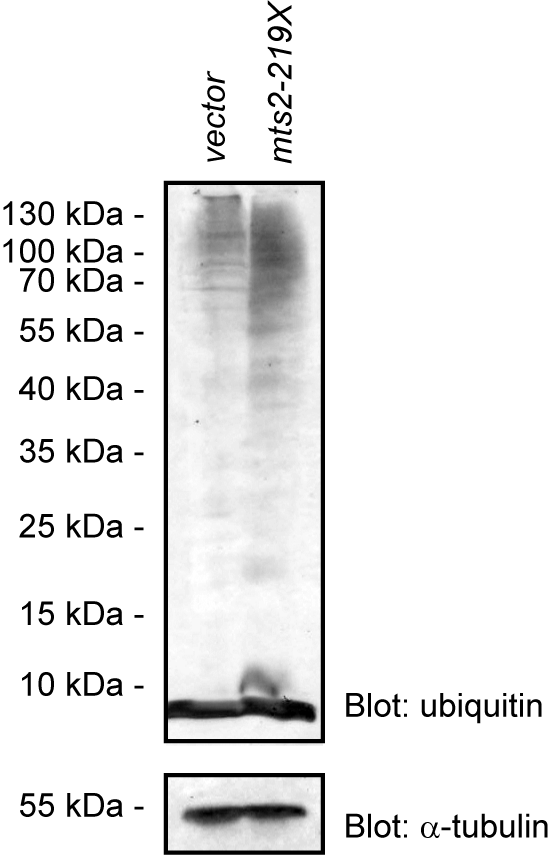

Supplement: Figure S6 — Ubiquitin-protein conjugates are stabilized upon expression of mts2-219X. Total cell extracts were analyzed for ubiquitin-protein conjugates by SDS-PAGE blotting with antibodies to ubiquitin. Tubulin served as a loading control. (TIF) [file pgen.1004140.s006.tif]
