# Supplementary material for: A Chaperone-Assisted Degradation Pathway Targets Kinetochore Proteins to Ensure Genome Stability
Source: PLoS Genet. 2014 Jan 30;10(1):e1004140. doi: 10.1371/journal.pgen.1004140 (PMC3907333; doi:10.1371/journal.pgen.1004140)
Supplement: Table S1 — Strains used. The fission yeast strains used in this study are listed. (DOC) [file pgen.1004140.s007.doc]

| **Table S1** | | |
| --- | --- | --- |
| Fission yeast strains used in this study | | |
| Strain | Genotype | Reference |
| *wild type* | *leu1-32 ura4-D18* | Lab. stock |
| *pad1-ZZ* | *pad1/rpn11- ProtAZZ, leu1-32 ura4-D18* | [1] |
| *bag101*Δ | *bag101::ura4 leu1-32 ura4-D18* | This study |
| *bag102*Δ | *bag102::arg3 leu1-32 ura4-D18* | This study |
| *spc7-GFP* | *spc7-GFP(G418) leu1-32 ura4-D18* | [2] |
| *spc7-23-GFP* | *spc7-23-GFP(G418) leu1-32 ura4-D18* | [2] |
| *spc7-23-GFP* | *spc7-23-GFP(NAT) leu1-32 ura4-D18* | This study |
| *mal2-1* | *mal2-1 leu1-32 ura4-D18* | [3] |
| *mis6-302* | *mis6-302 leu1-32 ura4-D18* | [4] |
| *mts2-1* | *mts2-1 leu1-32 ura4-D18* | [5] |
| *nas6*Δ | *nas6::G418 leu1-32 ura4-D18* | [6] |
| *ubc1*Δ | *ubc1::ura4 leu1-32 ura4-D18* | [7] |
| *ubc4-1* | *ubcP1-P61S(ura4^+^) leu1-32 ura4-D18* | [7] |
| *ubc6*Δ | *ubc6::ura4 leu1-32 ura4-D18* | [7] |
| *ubc7*Δ | *ubc7::ura4 leu1-32 ura4-D18* | [7] |
| *ubc8*Δ | *ubc8::ura4 leu1-32 ura4-D18* | [7] |
| *ubc11-1* | *ubcP4-140(ura4^+^) leu1-32 ura4-D18* | [7] |
| *ubc13*Δ | *ubc13::ura4 leu1-32 ura4-D18* | [7] |
| *ubc14*Δ | *ubc14::ura4 leu1-32 ura4-D18* | [7] |
| *ubc15*Δ | *ubc15::ura4 leu1-32 ura4-D18* | [7] |
| *ubc16*Δ | *ubc16::HYG leu1-32 ura4-D18* | [7] |
| *ubr1*Δ | *ubr1::ura4 leu1-32 ura4-D18* | [7] |
| *ubr11*Δ | *ubr11::ura4 leu1-32 ura4-D18* | [7] |
| *ubr11*Δ | *ubr11::G418 leu1-32 ura4-D18* | [6] |
| *ubp3*Δ | *ubp3::G418 leu1-32 ura4-D18* | [6] |
| *san1*Δ | *san1::G418 leu1-32 ura4-D18* | [6] |
| *hul5*Δ | *hul5::G418 leu1-32 ura4-D18* | [6] |
| *hrd1*Δ | *hrd1::G418 leu1-32 ura4-D18* | [6] |
| *doa10*Δ | *doa10::G418 leu1-32 ura4-D18* | [6] |
| *bag101*Δ *bag102* Δ | *bag101::ura4 bag102::arg3 leu1-32* | This study |
| *bag101*Δ *spc7-23-GFP* | *spc7-23-GFP(G418) bag101::ura4* | This study |
| *bag102*Δ *spc7-23-GFP* | *spc7-23-GFP(G418) bag102::arg3* | This study |
| *mts2-1 spc7-23-GFP* | *spc7-23-GFP(G418) mts2-1 leu1-32* | This study |
| *nas6*Δ *spc7-23-GFP* | *spc7-23-GFP(NAT) nas6::G418 leu1-32* | This study |
| *ubc1*Δ *spc7-23-GFP* | *spc7-23-GFP(G418) ubc1::ura4 leu1-32* | This study |
| *ubc4-1 spc7-23-GFP* | *spc7-23-GFP(G418) ubcP1-P61S(ura4^+^)* | This study |
| *ubc6*Δ *spc7-23-GFP* | *spc7-23-GFP(G418) ubc6::ura4 leu1-32* | This study |
| *ubc7*Δ *spc7-23-GFP* | *spc7-23-GFP(G418) ubc7::ura4 leu1-32* | This study |
| *ubc8*Δ *spc7-23-GFP* | *spc7-23-GFP(G418) ubc8::ura4 leu1-32* | This study |
| *ubc11-1 spc7-23-GFP* | *spc7-23-GFP(G418) ubcP4-140(ura4^+^)* | This study |
| *ubc13*Δ *spc7-23-GFP* | *spc7-23-GFP(G418) ubc13::ura4 leu1-32* | This study |
| *ubc14*Δ *spc7-23-GFP* | *spc7-23-GFP(G418) ubc14::ura4 leu1-32* | This study |
| *ubc15*Δ *spc7-23-GFP* | *spc7-23-GFP(G418) ubc15::ura4 leu1-32* | This study |
| *ubc16*Δ *spc7-23-GFP* | *spc7-23-GFP(G418) ubc16::HYG leu1-32* | This study |
| *ubr1*Δ *spc7-23-GFP* | *spc7-23-GFP(NAT) ubr1::ura4 leu1-32* | This study |
| *ubr11*Δ *spc7-23-GFP* | *spc7-23-GFP(G418) ubr11::ura4 leu1-32* | This study |
| *ubp3*Δ *spc7-23-GFP* | *spc7-23-GFP(NAT) ubp3::G418 leu1-32* | This study |
| *san1*Δ *spc7-23-GFP* | *spc7-23-GFP(NAT) san1::G418 leu1-32* | This study |
| *hul5*Δ *spc7-23-GFP* | *spc7-23-GFP(NAT) hul5::G418 leu1-32* | This study |
| *hrd1*Δ *spc7-23-GFP* | *spc7-23-GFP(NAT) hrd1::G418 leu1-32* | This study |
| *doa10*Δ *spc7-23-GFP* | *spc7-23-GFP(NAT) doa10::G418* | This study |
| *san1*Δ *mal2-1* | *san1::G418 mal2-1 leu1-32 ura4-D18* | This study |
| *san1*Δ *mis6-302* | *san1::G418 mis6-302 leu1-32 ura4-D18* | This study |
| *ubr11*Δ *mal2-1* | *ubr11::G418 mal2-1 leu1-32 ura4-D18* | This study |
| *ubr11*Δ *mis6-302* | *ubr11::G418 mis6-302 leu1-32 ura4-D18* | This study |
| *bag102*Δ *mal2-1* | *bag102::arg3 mal2-1 leu1-32 ura4-D18* | This study |
| *bag102*Δ *mis6-302* | *bag102::arg3 mis6-302 leu1-32 ura4-D18* | This study |
| *ubc4-1 mal2-1* | *ubcP1-P61S(ura4^+^) mal2-1 leu1-32 ura4-D18* | This study |
| *ubc4-1 mis6-302* | *ubcP1-P61S(ura4^+^) mis6-302 leu1-32 ura4-D18* | This study |
| *V5-ubr11* | *ubr11::V5-ubr11(G418) ura4-D18* | [8] |

**References to Table S1**

1. Stone M, Hartmann-Petersen R, Seeger M, Bech-Otschir D, Wallace M, Gordon C (2004) Uch2/Uch37 is the major deubiquitinating enzyme associated with the 26S proteasome in fission yeast. J Mol Biol 344: 697-706. S0022-2836(04)01214-8 [pii];10.1016/j.jmb.2004.09.057 [doi].

2. Kerres A, Jakopec V, Fleig U (2007) The conserved Spc7 protein is required for spindle integrity and links kinetochore complexes in fission yeast. Mol Biol Cell 18: 2441-2454. E06-08-0738 [pii];10.1091/mbc.E06-08-0738 [doi].

3. Fleig U, Sen-Gupta M, Hegemann JH (1996) Fission yeast mal2+ is required for chromosome segregation. Mol Cell Biol 16: 6169-6177.

4. Takahashi K, Yamada H, Yanagida M (1994) Fission yeast minichromosome loss mutants mis cause lethal aneuploidy and replication abnormality. Mol Biol Cell 5: 1145-1158.

5. Gordon C, McGurk G, Dillon P, Rosen C, Hastie ND (1993) Defective mitosis due to a mutation in the gene for a fission yeast 26S protease subunit. Nature 366: 355-357. 10.1038/366355a0 [doi].

6. Kim DU, Hayles J, Kim D, Wood V, Park HO, Won M, Yoo HS, Duhig T, Nam M, Palmer G, Han S, Jeffery L, Baek ST, Lee H, Shim YS, Lee M, Kim L, Heo KS, Noh EJ, Lee AR, Jang YJ, Chung KS, Choi SJ, Park JY, Park Y, Kim HM, Park SK, Park HJ, Kang EJ, Kim HB, Kang HS, Park HM, Kim K, Song K, Song KB, Nurse P, Hoe KL (2010) Analysis of a genome-wide set of gene deletions in the fission yeast Schizosaccharomyces pombe. Nat Biotechnol 28: 617-623. nbt.1628 [pii];10.1038/nbt.1628 [doi].

7. Penney M, Samejima I, Wilkinson CR, McInerny CJ, Mathiassen SG, Wallace M, Toda T, Hartmann-Petersen R, Gordon C (2012) Fission yeast 26S proteasome mutants are multi-drug resistant due to stabilization of the Pap1 transcription factor. PLoS One 7: e50796. 10.1371/journal.pone.0050796 [doi];PONE-D-12-28145 [pii].

8. Fujiwara H, Tanaka N, Yamashita I, Kitamura K (2013) Essential role of Ubr11, but not Ubr1, as an N-end rule ubiquitin ligase in Schizosaccharomyces pombe. Yeast 30: 1-11. 10.1002/yea.2936 [doi].
